# Measuring young adolescent perceptions of relationships: A vignette-based approach to exploring gender equality

**DOI:** 10.1371/journal.pone.0218863

**Published:** 2019-06-27

**Authors:** Robert W. Blum, Grace Sheehy, Mengmeng Li, Sharmistha Basu, Omaima El Gibaly, Patrick Kayembe, Xiayun Zuo, Jose Ortiz, Kitty S. Chan, Caroline Moreau

**Affiliations:** 1 Department of Population Family and Reproductive Health, Johns Hopkins Bloomberg School of Public Health, Baltimore, Maryland, United States of America; 2 Deutsche Gesellschaft fur Internationale Zusammenarbeit (GIZ), New Delhi, India; 3 Assiut University, Assiut, Arab Republic of Egypt; 4 Kinshasa School of Public Health, Kinshasa, Democratic Republic of the Congo; 5 Shanghai Institute of Planned Parenthood Research, Shanghai, China; 6 Faculty of Medical Sciences of the University of Cuenca-Ecuador, Cuenca, Ecuador; 7 Department of Health Policy and Management, Johns Hopkins Bloomberg School of Public Health, Baltimore, Maryland, United States of America; La Inmaculada Teacher Training Centre (University of Granada), SPAIN

## Abstract

This paper reports the development and baseline data of a vignettes-based measure of gender equality. *Methods*: Vignettes were developed through 3-day long focus groups. After piloting in 13 sites and repiloting a revised version in 6 countries, responses were categorized by the construct tapped and a scoring system developed. Finalized vignettes were then tested in DR Congo, Ecuador and China. *Results*: Young adolescents can successfully respond to vignettes; and can differentiate self from hypothetical protagonists of same and opposite sex. Response differences by sex of respondent and protagonist were statistically significant across a range of scenarios and settings. *Conclusion*: This is the first vignettes-based measure for young adolescents assessing young adolescent perceptions of relationships differentiated by sex of the protagonist.

## Introduction

Early adolescence (ages 10–14) is a critical period in the life course, as the onset of puberty and subsequent sociocultural practices and norms may increase engagement with a range of sexual and reproductive health experiences and outcomes, such as the initiation of romantic and sexual relationships[1,2]. With the onset of adolescence comes increased expectations of adherence to socially constructed gender norms that are learned and reinforced by adults as well as peers and reenacted as adolescents define their identity. It is during this stage of development that gender norms as well as associated gendered behaviors become increasingly rigid and enforced [3]. Girls, in particular, bear many of the consequences of gendered inequities, especially as their mobility often becomes more restricted than that of boys [4,5]. Exploring adolescents’ perceptions of gender norms is important for understanding how they perceive, experience and reenact their gender according to social scripts. Thus, understanding how young boys and girls relate to each other according to differential gendered scripts is critical for understanding the ways gender informs relationships. However, measurement of people’s perceptions about gender norms and how they regulate/constrain their daily lives has long posed methodological challenge to researchers [6–8].

Most studies assessing gender equality rely on direct questioning about an individual’s attitudes regarding differential traits, roles or outcomes attributed to each sex with specific emphasis on sexual and reproductive behaviors and outcomes (for gender equity and equality scales) [9]. While these gender attitudinal measures are valuable in predicting sexual risks in older adolescents and adult populations [10], they have limited applicability to younger populations, who have rarely engaged in sexual relations. At a more conceptual level, attitudinal measures focus on individual perceptions rather than on collective norms and are prone to error and social desirability bias, particularly for topics considered sensitive or too subtle for direct questioning. This is particularly a concern for exploring gender equality in early adolescence, as gender is both pervasive but abstract and therefore hard to conceptualize for this age group. To address these limitations, vignettes anchor the exploration of gender equality by grounding the exploration of gender equality in concrete stories about relationships. In these scenarios adolescents are first asked to evaluate a situation where a boy is in the lead and then subsequently, using the same scenario, when a girl is the lead. Additionally, by asking how a situation would typically be resolved by adolescents and how the adolescent respondent would personally react to the situation it becomes possible to capture both individual attitudes and collective norms.

### Use of vignettes

The vignette methodology is a research tool that has been used to assess attitudes, values, norms and perceptions, particularly regarding sensitive topics in the health and social sciences [8,11] and especially with qualitative research [12]. For example, Spratt [13] built vignettes based on child protection cases to explore service provider attitudes. Vignettes often take the form of stories or narratives about people or situations. These stories are presented to participants who are asked how they or the central character (protagonist) might respond to the situation [11]. Vignette narratives may be generated through prior or formative research, collaborative efforts, or true stories [11].Vignettes are useful in that they clearly outline the situation under study, setting the same context for all participants, thus ensuring comparability, while also providing more opportunity for interpretation than traditional survey methods [8].

Vignettes allow participants to express their opinions in a non-threatening manner, usually by asking respondents to indicate what they think others in such a situation *would do* (e.g., descriptive norms) but it is equally feasible to assess what respondents think others *should do* (injunctive norms). Such an approach does not require the respondent to indicate what they themselves would do thereby reducing the pressure to provide socially desirable responses or to feel excessively self-revealing [8]. This makes vignettes especially valuable for topics including gender norms, gender discrimination, drug use, mental illness, and emotional and behavioral difficulties [6,14–18]

A criticism of vignettes is that they do not truly reflect real life due, in part, to the very distance they create from the narrative that facilitates participants’ unvarnished responses. However, Hughes [11] points out that no quantitative methodology can truly reflect real life experiences; and thus, as with any other method, researchers need to acknowledge the limitations of vignettes. Importantly, however, studies have found that people often respond to vignettes similarly to how they respond to situations in real life [11]. Since the researcher does not know the respondent’s reality [11,19], careful vignette construction is essential to ensure that they are relevant, realistic, and engaging for participants [20].

Vignettes have additional promise when conducting research with children and adolescents. Studies using vignettes with young people have explored topics including nutrition, cultural tolerance, sexual health and relationships [21–23]. Vignettes are a useful tool for engaging young people, especially in research on sensitive subjects and abstract concepts, as they allow for youths’ active participation and control throughout the process [21,24].

A primary challenge of using a vignette methodology is in interpreting responses to vignettes, as participants may shift between speaking about themselves and the character [8,25]. With respect to research on adolescents, there are further challenges in ensuring vignettes are tailored to their experiences, language and contexts. Additionally, there is limited experience using vignettes in low and middle income countries since this methodology has primarily been used in the Global North [19,26].

### Instrument development for the Global Early Adolescent Study

The Global Early Adolescent Study (GEAS) is a multi-country study among adolescents aged 10–14 that explores the relationships between gender norms and adolescent health and wellbeing (sexual and reproductive health, mental health, gender-based and interpersonal violence, school retention and healthy sexuality) cross-culturally and across time. The development and implementation of the study began with a narrative process of listening to the voices of young people in 15 countries globally and has been reported in detail elsewhere [27]. Subsequently, the study involved the development and piloting of several instruments with approximately 120 young people in each of 14 sites in an equal number of countries on 5 continents, including Shanghai, China; Hanoi, Viet Nam; New Delhi, India; Assuit, Egypt; Nairobi, Kenya; Blantyre, Malawi; Cape Town, South Africa, Kinshasa, DR Congo; Ile Ife, Nigeria; Ouagadougou, Burkina Faso, Dar Es Salaam, Tanzania; Ghent, Belgium; Edinburgh, UK; Cuenca, Ecuador, Cochabamba, Bolivia; Baltimore, United States. After reanalysis, all measures were revised and repiloted with 75 young people equally divided between the sexes and across 10 to 14 year olds. Three instruments were finally developed including: a measure of gender norms, a 10-module measure of health and social contexts and a vignettes measure of gender equality in relationships. The vignettes measure assesses how young people in a community think about situations differently if a boy or girl is in the lead. As such it measures the gap in perceptions that boys and girls express depending on the sex of the protagonist. The development of the vignettes measure for early adolescents is the focus of this paper.

## Materials and methods

### Vignette development

Vignettes were initially developed through three-day long focus group discussions and role-plays with young adolescents in each of the participating sites, and were based on common situations young people identified. The goal was to generate scenarios that were as close to real-life as possible. Each collaborating site was asked to select between 10 and 12 young people ages 11–14 years. The criteria for selection were that they were verbal and literate, represented both boys and girls in approximately equal proportions, and had parental permission and the acceptance of the adolescents themselves to participate for three days. In all sites mixed sex groups of young people participated. Generally, the groups met for three consecutive days but in some settings two sessions were held for a day-and-a-half each over two consecutive weekends. At each site, there was a facilitator and at least two researchers/ research assistants whose job was to keep detailed notes and subsequently to convert the discussions into vignettes with questions, and response options (vignette measures are included in S3 Text).

Initially, participants were asked to generate a list of common situations that they and their peers experienced in school, community, family, and other social contexts. Subsequently, they were asked to rank order the situations as to the ones they felt to be most common and important to discuss. The intent was to generate approximately six vignettes in each site over three days.

Once ranking was completed the facilitator led an open discussion about the top-ranked situation. Participants were asked to elaborate details about that situation. The central characters of the situation were given names by the participants; these names remained constant throughout all of the scenes. Two young people were asked to role play the situation. After approximately five minutes the facilitator stopped the role-play and asked the two “actors” to debrief from their roles. This was followed by a general discussion where other members of the group were invited to comment on alternative ways the situation could play out and other possible conclusions.

Subsequently, the sex of the lead characters was reversed. In this way, for every vignette there were male and female protagonists. At the conclusion of role-plays, discussion and debriefing there was a break with snacks. The process was repeated throughout the workshop. During lunch and evenings, the researchers compiled notes and generated vignette stems with a storyline developed by the young people and on the morning of the next day the draft vignettes were distributed to the youth participants who critiqued everything from language to the framing of the scenario segments and the response options. In this way, the vignettes that were developed reflected the thinking, reality and language of the youth participants. Vignettes developed in local language were then translated into English by country collaborators proficient in both English and the local language. The translated English version was then refined by the research team in Baltimore. The revised version was subsequently back-translated to each local language for further proofreading to ensure content closely reflect the reality in local culture. Using an iterative process, a final English version was developed.

### Developing common vignettes across sites and initial piloting

At the conclusion of the three days the compiled vignettes were sent to the Hopkins Coordinating Centre where a content analysis was done once all sites submitted their vignettes. After extensive discussion with global research partners, six common vignettes were identified for piloting across sites exploring the following themes: romantic interests, freedom of movement, wearing clothes parents felt to be inappropriate for adolescents, reactions to puberty, issues of responsibility surrounding causing a pregnancy/ becoming pregnant, and gender-atypical behavior of a peer. As noted above, the original vignettes were piloted along with a more extensive set of quantitative measures in 13 sites with a convenience sample of 120 young people in each site (except Nairobi with a sample just under 400) all of whom were between the ages of 10 and 14 years (vignettes measure in initial piloting are available in S3 Text). Two sites—Baltimore and Edinburgh—did not participate. After piloting four vignettes were retained. The two that were dropped dealt with appropriate clothing and freedom of movement; and they were dropped because there were insufficient variations to response options (deleted vignette measures are presented in S2 Text).

### Repilot

The four revised vignettes (exploring romantic interests, issues of responsibility for causing a pregnancy/becoming pregnant, reacting to puberty, and responding to gender atypical behavior of a peer) were re-piloted with approximately 75 young people in each of 6 sites (Ghent, Belgium; Assiut, Egypt; Cuenca, Ecuador; Blantyre, Malawi; Hanoi, Viet Nam; Kinshasa, Democratic Republic of the Congo) proportionately divided in each site by sex and age from 10–14 years. For the repilot, respondents predominantly answered same sex vignettes; however, one vignette (gender atypical behavior) required respondents to also take the perspective of the opposite sex. Additionally, in the repilot a limited number of questions were added that assessed what respondents thought they would do in the same situation (vignette measures in repilot are included in S4 Text).

### Universal application but site specific

While the core meaning of each vignette was retained across geographies, the situations were modified to be appropriate for each country’s context. So, for example, in one setting the vignette on romantic relationships was built around young people attending a dance while in another they were going to a wedding party. To navigate cultural variation across sites, the response options were developed through iterative discussions among all partners to reach consensus on the meaning of each response and remove any ambiguity, and response choices remained fixed across settings. Vignettes development, piloting, re-piloting, and their inclusion in cross-site baseline surveys were all carried out among socioeconomically disadvantaged populations of young adolescents in each site based on area of residency.

### Field coordinator survey

Supplementing the repilot, a survey was conducted among the 13 field coordinators from all sites to assess their experiences developing and administering vignettes and to identify issues in administration. A semi-structured questionnaire was distributed among all the original sites with nine (60%) responding. We used a matrix to organize data and facilitate identification of primary themes [28] (field coordinator survey is accessible in S5 Text).

### Scoring of vignettes

For scoring and subsequent analysis of vignette pilot data, response options were first characterized along seven potential domains including: communication style, interactive approach, assertiveness, social inclusion, peer attitudes toward gender atypical behaviors, emotional response to puberty and pregnancy responsibility. The higher the score the more positive was the endorsement of response (e.g., more direct the communication, more assertive) (Table 1). Detailed scoring for vignettes measure responses can be found in S1 Table.

**Table 1:** Score system for seven core domains across three sites.

Core Domain	Score System
**Communication**	0: Avoidance
	1: Indirect
	2: Direct
**Assertiveness**	0: No
	1: Low
	2: Moderate
	3: High
**Interaction Approach**	0: Avoidance
	1: Antagonist initiates
	2: Protagonist initiates
**Social Inclusion**	0: No
	1: Some
	2: Yes
**Peer Attitudes toward Gender Atypical Behaviors**	0: High (most aversive)
	1: Moderate-high
	2: Low-moderate
**Emotional Response to Puberty**	0: Very negative
	1: Negative
	2: Neutral
	3: Positive
**Pregnancy Responsibility**	0: Deny responsibility
	1: Accept responsibility

The final version of the vignettes was produced in Baltimore in English, and translated into the local language and vernacular by each collaborating site.

Following the formative stage of GEAS, a second phase of the GEAS consists in exploring longitudinally the ways in which gender norms evolve from early to later adolescent years and how these norms relate to behavioral and health outcomes over time. From June 2017 to March, 2018, phase II baseline surveys were conducted in three sites: Kinshasa, DR Congo (N = 2847); Shanghai, China (N = 1776); Cuenca, Ecuador (N = 704). Phase II Baseline survey incorporated three of the four piloted vignettes (romantic interests, reacting to puberty, and gender-atypical behavior of a peer) in all three sites, whereas a vignette exploring issues of responsibility surrounding causing a pregnancy/ becoming pregnant was administered in two of the three sites (Kinshasa and Cuenca). The vignettes were the same as the re-piloted vignettes of phase 1, with the exception of the romantic interests vignette which was expanded to include questions both reversing the sex of the protagonist as well as same sex respondent-protagonist to directly capture respondent differences in attitudes for when a boy or a girl was in the lead.

### Data analysis

Adolescents with complete responses to all vignettes were included in the analysis with a sample size of 2586 for Kinshasa, 1645 for Shanghai, and 484 for Cuenca. Site-specific demographic characteristics are available in S2 Table. Response domains were summarized based on the interpretation of each vignette question (e.g. communication, assertiveness, social inclusion, emotional response to puberty and pregnancy). A scoring system was developed to quantify responses to each domain across vignettes. For example, the communication domain included one question assessing how a boy or girl protagonist would get attention from the individual of his or her romantic interests. Based on the given responses, a score ranging from 0 to 2 was assigned correspondingly to each option (0: Avoidance, 1: Indirect, 2: Direct). Specifically, a score of 0 was given if the option selected was that the respondent would take no action to increase the likelihood of that occurring, a score of 1 for choosing to ask friend to help communicate the self-romantic interests to the individual, score 2 for choosing to directly contact the individual by passing on a note or in-person communication. Mean scores at group level per domain by sex per site were calculated and compared by Student t-test or Wilcoxon rank-sum test as appropriate depending on the normality of score distribution and homogeneity of variance. Mean scores at the individual level for paired questions were performed by paired Student t-tests or Wilcoxon matched-pairs signed-ranks tests as appropriate. Two-sided p-values of less than 0.05 were considered statistically significant. All analyses were conducted using Stata Version 14.2 (StataCorp LLC, College Station, TX).

We used two ways of approaching analyses of gender equality. We consistently compared average scores for boy and girl respondents by site for all domains. That being said, for vignettes without a gender-flipping design, which did not allow comparison at the individual level, we tested the mean scores by sex as a proxy for gender equality at the population level. For some vignettes (e.g. social inclusion of gender atypical peers and romantic interests) with flipped gender perspective design to assess gender quality, we compared same versus opposite gender perspective scores from the same participant on an individual level.

### Ethical review

The study protocol was reviewed and approved by the World Health Organization’s Ethical Review Board (ERC#2638) the Johns Hopkins Bloomberg School of Public Health Institutional Review Board (IRB#5684) and each of the countries’ institutional review boards. In each site, active parental consent was obtained as was participant assent.

## Results

### Domains where the sex of protagonist was flipped

In this section, we present findings on the ability of young adolescents to take the perspective of the opposite sex in reference to scenarios in which the gender of the protagonist “flips” (Table 2). Four domains that tap gender norms differences will be explored in depth: communication style, interactive approach, assertiveness, and emotional response to puberty. Finally, we explore the differences when descriptive norm questions are asked for self and other.

**Table 2:** Communication by gender flipped perspectives across sites (Vignette 1 –question 1 & 3 in S1 Text).

		Boys				Girls		
*Site (Mean*, *SD)*	*N*	*Male Protagonist*	*Female Protagonist*	P-value	*N*	*Male Protagonist*	*Female Protagonist*	P-value
**Kinshasa (DRC) (n = 2586)**	**1274**	1.61 (0.65)	1.53 (0.65)	<0.001^§^	**1312**	1.63 (0.64)	1.52 (0.73)	<0.001^§^
**Shanghai (China) (n = 1645)**	**830**	1.11 (0.90)	1.15 (0.87)	0.285^^^	**815**	1.51 (0.75)	1.07 (0.90)	<0.001^§^
**Cuenca (Ecuador) (n = 484)**	**258**	1.73 (0.57)	1.57 (0.70)	0.007^§^	**226**	1.68 (0.61)	1.52 (0.77)	0.049^§^

Note

^ = Paired Student t-test.

§ = Wilcoxon matched-pairs signed-ranks test.

#### Communication style

Vignettes relating to communication explored direct and indirect styles when approaching a member of the opposite sex. In Cuenca, there were statistically significant differences in how a boy would respond when taking the perspective of a male protagonist versus that of a female protagonist (p<0.01), and differences in how a girl would respond when flipping gender perspectives (p<0.05). Both boys and girls perceived that males would be more direct in communication style than females. In Kinshasa, there were statistically significant differences in how both boys and girls responded when taking the perspective of a protagonist of the same sex versus one of the opposite sex (p<0.001). Again, both groups perceived a male protagonist to be more direct in communication than a female protagonist. In Shanghai, girls were significantly more likely to perceive a male protagonist as direct than a female protagonist (p<0.001), while boys’ responses reflected no significant difference when taking a male versus a female perspective (p = 0.285).

#### Interaction approach

This domain includes vignettes relating to a character’s likelihood of initiating conversation with a member of the opposite sex (Table 3). In Cuenca, girls reacted significantly differently when responding as a girl character versus taking the perspective of a boy, who they perceived to be more outgoing (p<0.01). Boys responded no differently when taking the perspective of a girl or boy (p = 0.363). In Kinshasa, there were significant differences among both boy and girl respondents in how they responded to a scenario when taking the perspective of their own versus the opposite sex (p<0.001). While boys perceived girls as being more outgoing, girls perceived boys as being more outgoing. Meanwhile, in Shanghai girls perceived boys as being significantly more outgoing (p<0.01), while boys responded almost no differently when taking the perspective of a girl versus that of a boy.

**Table 3:** Interaction approach by gender flipped perspectives across sites (Vignette 1 –question 4 & 6, see S1 Text).

		Boys				Girls		
*Site (Mean*, *SD)*	*N*	*Male Protagonist*	*Female Protagonist*	P-value	*N*	*Male Protagonist*	*Female Protagonist*	P-value
**Kinshasa (DRC) (n = 2586)**	**1274**	1.55 (0.79)	1.76 (0.55)	<0.001^§^	**1312**	1.67 (0.68)	1.42 (0.85)	<0.001^§^
**Shanghai (China) (n = 1645)**	**830**	1.64 (0.56)	1.62 (0.59)	0.424^§^	**815**	1.71 (0.53)	1.64 (0.58)	0.008^§^
**Cuenca (Ecuador) (n = 484)**	**258**	1.71 (0.49)	1.69 (0.47)	0.363^§^	**226**	1.66 (0.56)	1.56 (0.56)	0.007^§^

Note

§ = Wilcoxon matched-pairs signed-ranks test.

#### Assertiveness

Vignettes within the assertiveness domain were scored from low to high in terms of how assertively a protagonist would approach a member of the opposite sex (Table 4). In all three settings, there were significant differences (p<0.04) between how both boys and girls would respond when taking the perspective of their own sex versus that of the opposite sex. Across the three settings, both sexes perceived boys as being significantly more assertive than girls.

**Table 4:** Assertiveness by gender flipped perspectives across sites (Vignette 1 –question 5 & 7, see S1 Text).

		Boys				Girls		
*Site (Mean*, *SD)*	*N*	*Male Protagonist*	*Female Protagonist*	P-value	*N*	*Male Protagonist*	*Female Protagonist*	P-value
**Kinshasa (DRC) (n = 2586)**	**1274**	2.34 (1.03)	2.19 (1.11)	<0.001^§^	**1312**	2.42 (0.99)	1.84 (1.21)	<0.001^§^
**Shanghai (China) (n = 1645)**	**830**	1.73 (1.26)	1.44 (1.20)	<0.001^§^	**815**	2.05 (1.23)	1.50 (1.28)	<0.001^§^
**Cuenca (Ecuador) (n = 484)**	**258**	2.54 (0.95)	2.38 (1.04)	0.032^§^	**226**	2.38 (1.06)	2.15 (1.19)	0.004^§^

Note

§ = Wilcoxon matched-pairs signed-ranks test.

#### Social inclusion

Vignettes within the social inclusion domain explored how accepting adolescents would be of a gender atypical peer (Table 5). In both Kinshasa and Shanghai, there were significant differences in adolescents’ responses when they were taking the perspective of a boy versus that of a girl (p<0.01). In Shanghai, both boys and girls perceived girls as being significantly more inclusive than boys. In contrast, in Kinshasa, boys perceived girls as being less inclusive than boys, while girls believed the opposite. In Cuenca, boys perceived their own sex as being significantly more inclusive, while girls perceived both sexes to be nearly equally inclusive.

**Table 5:** Social inclusion by gender flipped perspectives across sites (Vignette 2 –question 1 & 4, see S1 Text).

		Boys				Girls		
*Site (Mean*, *SD)*	*N*	*Male Protagonist*	*Female Protagonist*	P-value	*N*	*Male Protagonist*	*Female Protagonist*	P-value
**Kinshasa (DRC) (n = 2586)**	**1274**	0.58 (0.89)	0.49 (0.84)	0.001^^^	**1312**	0.32 (0.71)	0.69 (0.93)	<0.001^^^
**Shanghai (China) (n = 1645)**	**830**	0.86 (0.97)	1.23 (0.96)	<0.001^^^	**815**	0.77 (0.95)	1.09 (0.98)	<0.001^^^
**Cuenca (Ecuador) (n = 484)**	**258**	1.36 (0.92)	1.20 (0.95)	0.018^^^	**226**	1.15 (0.96)	1.13 (0.94)	0.820^^^

Note

^ = Paired Student t-test.

### Domains exploring sex differences in responses without flipping perspectives

Several domains asked participants to respond to questions for a same sex and age protagonist as themselves so as to explore issues surrounding pubertal change and attitudes to peers with atypical gendered behaviors.

#### Peer understanding towards gender stigma (Vignette 2—question 3)

In all three sites, there were significant differences between how boys and girls thought their peers would include or exclude a protagonist who acted atypically for his or her gender (for instance, a boy who preferred to play with girls asking a group of girls if he could play with them) (Table 6). In Cuenca and Shanghai, girl respondents anticipated that the same-sex peers of female protagonist would be more accepting than boy respondents thought the same-sex peers of male protagonist would be (p<0.05 and p<0.01, respectively). Meanwhile, in Kinshasa boys believed the same-sex peers of a male protagonist would be more accepting of a gender atypical girl than girl respondents believe a group of girls would be of a gender atypical boy (p<0.01).

**Table 6:** Vignettes measures for unpaired questions across sites.

	Kinshasa (DRC) (n = 2586)			Cuenca (Ecuador) (n = 484)			Shanghai (China) (n = 1645)		
*Domain (Mean*, *SD)*	*Boys (n = 1274)*	*Girls (n = 1312)*	P-value	*Boys (n = 258)*	*Girls (n = 226)*	P-value	*Boys (n = 830)*	*Girls (n = 815)*	P-value
***Peer Understanding (toward gender stigma)***	0.53 (0.50)	0.46 (0.50)	**^^^	0.69 (0.46)	0.79 (0.41)	*^§^	0.63 (0.48)	0.75 (0.43)	***^^^
***Puberty***									
** Emotional Response**	2.22 (1.05)	2.13 (1.08)	*^^^	1.76 (1.12)	1.67 (1.02)	ns^^^	1.90 (1.00)	1.80 (0.91)	*^§^
** Proactive**	1.52 (0.79)	1.75 (0.55)	***^§^	1.73 (0.52)	1.87 (0.41)	***^§^	1.55 (0.67)	1.76 (0.54)	***^§^
** Parent Response**	0.73 (0.45)	0.57 (0.50)	***^§^	0.96 (0.20)	0.97 (0.16)	ns^^^	0.97 (0.17)	0.95 (0.21)	ns^§^
** Peer Response**	0.63 (0.74)	0.54 (0.65)	**^^^	0.84 (0.79)	0.74 (0.66)	ns^^^	0.80 (0.62)	0.80 (0.51)	ns^§^
***Pregnancy***							Not Applicable		
** Responsibility (respondent perspective)**	0.52 (0.50)	0.44 (0.50)	***^^^	0.51 (0.50)	0.41 (0.49)	*^^^			
** Parental Support**	0.72 (0.45)	0.70 (0.46)	ns^^^	0.71 (0.45)	0.87 (0.34)	***^§^			
** Peer Responsibility**	0.56 (0.50)	0.55 (0.50)	ns^^^	0.63 (0.48)	0.72 (0.45)	*^^^			

Note

^ = Student t-test

§ = Wilcoxon rank-sum test. ns = no statistical significance.

*p<0.05

**p<0.01

***p<0.001

#### Responses to puberty (Vignette 3—question1, 3, 4)

Another vignette explored responses to puberty and specifically how an individual, their parents and their peers might respond to their visible onset of puberty (Table 6).

In both Kinshasa and Shanghai boy respondents consistently reported (through the lens of male protagonists) to have more satisfaction and pride with pubertal body changes than the girl respondents (both p<0.05). However, in Cuenca, there were no statistically significant differences in responses between boys and girls which may be the consequence of a relatively smaller sample size. Conversely, in all three study sites girls reported that the female protagonist would be more proactive in seeking answers to questions she would have than boys thought the male protagonist would be (p<0.001).

When asked how they thought their parents would respond to the onset of puberty, responding through a same sex avatar, only in Kinshasa did boys believe their parents would respond more positively to their physical changes than girls (0.73 vs 0.57; p<0.001). In both Cuenca and Shanghai, both boys and girls believed that their parents would respond equally positively to their maturation. A similar finding was seen when boys and girls were asked how peers would respond to the pubertal onset of the same sex avatar; specifically, they thought that there would be no differential gendered responses by male or female peers. Only in Kinshasa did boys perceive a more positive response for male protagonist puberty than girls did for the female one (p<0.01).

#### Responses to pregnancy (Vignette 4—question 3, 5, 7)

A final series of questions in the pregnancy vignette explored responses to pregnancy, both in terms of how an individual boy or girl would respond to causing or becoming pregnant, respectively, and how their parents and peers might respond (Table 6). In both Kinshasa and Cuenca, there were statistically significant sex differences with boys being more likely to report themselves as taking responsibility for a pregnancy than girls did (Kinshasa: 0.52 vs. 0.44, p<0.001; Cuenca: 0.51 vs. 0.41, p<0.05) but there were no differences seen when they responded for a same sex protagonist. In Kinshasa, both boys and girls perceived parents as being fairly supportive (0.72 and 0.70 on a scale of 0–1) of an adolescent’s pregnancy; in Cuenca, girls thought parents would be more supportive than boys did (0.87 vs. 0.71, p<0.001). In Cuenca, there was a small but statistically significant difference in how participants imagined same sex peers would respond to a pregnancy, with girls imagining their peers as more likely to accept responsibility than boys did theirs (p = 0.047). No sex differences were seen in Kinshasa in relationship to pregnancy response.

#### Pregnancy responsibility (Vignette 4—question 2, 3)

In both Kinshasa and Cuenca, when asked about assuming responsibility for having caused the hypothetical pregnancy, boys indicated that they personally would be more likely to assume responsibility than the avatar (p<0.001). For girls, the difference was significant only in Kinshasa (p<0.01) where like their male peers they thought that they would assume more responsibility for the pregnancy than their female protagonist.

### Domains where self and same sex protagonist were compared

#### Communication (Vignette 1—question 1, 2)

The same vignette was used to assess communication style adopted by adolescents when they would like to approach an individual of the opposite sex (Table 7). Assessment was carried out first by projecting the respondent into protagonist perspective and then the perspective of self. In Cuenca, there was a statistically significant difference in how girls responded to scenarios when they were speaking from their own perspective versus that of a hypothetical character (p<0.03); overall, girls perceived the character as being more direct than they themselves would be. In contrast, there was no statistically significant difference in how boys responded for themselves and the male protagonist (p = 0.347). In Kinshasa, when we compared the level of direct communication (e.g. communication style that would be taken when a boy protagonist was attracted to a girl and wanted to get her attention and vice versa) for themselves compared with a same sex protagonist both boys and girls thought the protagonist would be significantly more direct than they would be (p<0.001). The same was seen in Shanghai (p<0.001).

**Table 7:** Communication by self versus protagonist perspectives across sites.

		Boys			Girls		
*Site (Mean*, *SD)*	*N*	*Self*	*Male Protagonist*	P-value	*N*	*Self*	*Female Protagonist*	P-value
**Kinshasa (DRC) (n = 2586)**	**1274**	1.49 (0.77)	1.61 (0.65)	<0.001^§^	**1312**	1.18 (0.90)	1.52 (0.73)	<0.001^§^
**Shanghai (China) (n = 1645)**	**830**	0.70 (0.91)	1.11 (0.90)	<0.001^§^	**815**	0.60 (0.88)	1.07 (0.90)	<0.001^§^
**Cuenca (Ecuador) (n = 484)**	**258**	1.68 (0.65)	1.73 (0.57)	0.347^§^	**226**	1.41 (0.86)	1.52 (0.77)	0.027^§^

Note

§ = Wilcoxon matched-pairs signed-ranks test.

#### Field coordinator perspectives

Field coordinators (FC) offered a range of insights on the process of developing and administering vignettes, including challenges faced and recommendations for other researchers using this methodology. Fourteen FC were surveyed; and nine responses were received.

#### Challenges faced during the vignette workshop

Three of the nine respondents felt that the duration of the workshop was a challenge as interest and interaction would decline after lunch; and multiple breaks were required to keep adolescents engaged. Additionally, role plays with mixed groups (boys and girls) were challenging according to two sites as adolescents were shy to engage with the opposite sex. Other challenges reported related to: security concerns (groups of young people tried to break into the places where the sessions were going on), risk of equipment theft and thus a need for guards in certain sites, obtaining parent permissions and finding appropriate venues.

#### Lessons learned and recommendations

Recommendations centered around a) workshop duration and venue, and b) participants and their interactions. Three respondents suggested limiting sessions to half a day believing that would increase engagement. Additionally, coordinators felt that spaces with outdoor recreational opportunities for breaks increased youth participation. Some respondents felt that while they would not change the mixed-sex nature of the groups, it proved to be challenging at times; as one respondent noted: “*Mixed group sessions (boys and girls together) is the best option for capturing gender biases or debates for generating more options for a stem”* [New Delhi, Field Coordinator]. Role plays were also an integral part of the vignettes as was the opportunity for youth participants to critique and revise vignette drafts. As the Shanghai Field coordinator noted: “*Critique and validity by adolescents of each vignette was important*, *including the role play*. *The plot proceeding and other choices were revised and clarified during the critique by adolescents”*.

Another recommendation included expanding the number of note takers so as to better capture the dialogue since too often the facilitator also became one of the primary note-takers (additional field coordinator survey findings are summarized in S6 Text).

## Discussion

This paper presents the first measure using vignettes to assess gender equality for boys and girls developed specifically for early adolescents across low and middle as well as high income countries. Findings from our baseline data in three settings demonstrate that young adolescents can successfully respond to vignettes; and likewise, they can differentiate self from a hypothetical protagonist both of the same and opposite sex. With a few exceptions, differences between responses by sex of respondent and that of protagonist were statistically significant across a range of scenarios in all settings.

Vignettes present both a feasible and interactive means of engaging young people in data collection in a manner that keeps them engaged and incorporates their own stories and experiences into the research process. Further, vignettes in the present measure were developed by young people and thus were relevant to the experiences of participants in each of the settings.

Additionally, across the three sites, both boys and girls were able to differentiate between male and female protagonists when responding to vignettes. Across the four domains that tap relationships, there were statistically significant differences in how both girls and boys responded when taking the perspective of someone of the same and then the opposite sex; thereby, allowing for assessment of both individual and group gender norms about relationships.

Overall, vignettes were useful for quantifying differences between boys and girls in communication approaches, social inclusion, interpersonal styles and acceptance of gender atypical peers. Vignettes allow us to explore population differences between boys and girls and to tap how each group thinks about differences when a boy or girl is in the lead in situations common to their age group. So too, the preponderance of evidence from the analyses of vignettes data from the three initial sites in the DR Congo, China and Ecuador suggest that young adolescents are generally able to distinguish self from a protagonist and are likewise able to take the perspective of the opposite sex when answering questions about specific situations depicted in the vignettes. However, such perspective taking may vary by vignette and context. For example, Swartzman & McDermid [29] found that college students reported difficulty in taking the perspective of a vignette character who was much older than them.

The vignette methodology can reduce response bias when asking direct questions through the lens of a hypothetical protagonist, and allows for assessing gender attitudes at multiple levels. By using focus groups across geographically and culturally diverse sites the vignettes developed reflect the realities of the daily lives of young people. Likewise, as respondents to the vignettes young people are more actively engaged with situations than is possible with more traditional survey methods. Similar to other studies (8) we also found vignettes useful in minimizing social desirability bias by allowing participants to take the perspective of a different character in discussing potentially sensitive topics. Issues related to sexuality and romantic interest were woven throughout several vignettes, and were found to be well-suited to the developmental phase of participants. Vignettes were useful in allowing us to tap into different dimensions of gender norms (i.e. communication style, interaction approach, assertiveness) than those that are captured in more traditional measures of gender equality, for example, in work or education.

The challenges to vignette development for early adolescents included: the young age of participants, most all of whom are still in the process of developing abstract thinking abilities; the social, cultural and linguistic diversity in the settings where we worked; and the complexity of measuring an abstract concept like gender equality. Development of the vignettes was a time-consuming process that took place over one year and numerous vignettes versions were generated before the final set of five emerged.

Given the few studies that have been conducted on use of vignettes in low and middle-income countries, this research and instrument fills an important gap.

### Limitations

A possible limitation of this work could be our lack of open-ended questions to obtain adolescents’ rationale for response choices. However, the vignettes measure was developed to be administered in survey format; and the aim was to measure gender attitudes and not predict real life behavior. Additionally, these vignettes were used only among the poorest quintile of young adolescents in each setting; responses may be different with a more diverse socioeconomic population. Likewise, rural and peri-urban young people were not part of the sample; and a more diverse population may have yielded different results.

## Conclusion

This is the largest study of young adolescents ever undertaken across diverse cultures and geographies to test the development of a vignettes- based measure of gender equality in relationships. The results, while not definitive, are encouraging and warrant further exploration and validation against other gender norms measures. If subsequent research sustains these findings, vignettes will be a valuable tool for assessing gender norms; and for those who do gender norm change programming, vignettes may prove to be a useful adjunct to assess change in gender norms as they apply to interpersonal relationships over time.

## Supporting information

S1 Table Vignette Example and corresponding scoring system. (DOCX)

S2 Table Demographic characteristics for analytical sample by site. (DOCX)

S1 Text Vignette-based measures of gender equality. (DOCX)

S2 Text Dropped vignette measures during vignettes development. (DOCX)

S3 Text Vignettes instrument (Pilot)—The Global Early Adolescent Study. (DOCX)

S4 Text Vignettes instrument (Repilot)—The Global Early Adolescent Study. (DOCX)

S5 Text The GEAS Field Coordinator Survey. (DOCX)

S6 Text Summary of additional detail on the Field Coordinator Survey. (DOCX)
