# Supplementary material for: Measuring young adolescent perceptions of relationships: A vignette-based approach to exploring gender equality
Source: PLoS One. 2019 Jun 27;14(6):e0218863. doi: 10.1371/journal.pone.0218863 (PMC6597075; doi:10.1371/journal.pone.0218863)
Supplement: S1 Table — (DOCX) [file pone.0218863.s001.docx]

| ***S1 Table: Vignette Example and Corresponding Scoring System*** | | |  |
| --- | --- | --- | --- |
| **Domain** | **Vignette** | **Response** | **Score System** |
|  | X is attracted to Y, but has never spoken with Y. X wants to get Y's attention, but is not sure how. Protagonist question: What do you think X would do to get Y's attention? Respondent question: What option would you choose in that situation? | Ask a friend to tell Y that X likes him/her. ***(Indirect)*** | 0: Avoidance 1: Indirect 2: Direct |
| ***Communication*** |  |  |  |
|  |  | Pass Y a note. ***(Direct)*** |  |
|  |  | Go up and talk to Y directly. ***(Direct)*** |  |
|  |  | Nothing, just wait, hoping to meet Y. ***(Avoidance)*** |  |
|  |  |  |  |
| ***Interaction Approach*** | It is Friday, and X and Y are both at a party with their friends. X (attracted to Y) sees that Y is standing in a corner across the room. Protagonist question: What would it take for X to talk to Y? | X is challenged by friends. ***(Protagonist initiates)*** | 0: Avoidance 1: Antagonist initiates 2: Protagonist initiates |
|  |  |  |  |
|  |  | Y was alone. ***(Protagonist initiates)*** |  |
|  |  | Y came up to X or noticed X in another way. ***(Antagonist initiates)*** |  |
|  |  |  |  |
|  |  | X knew from a friend that Y liked him/her. ***(Protagonist initiates)*** |  |
|  |  |  |  |
|  |  | X would not talk to Y under any circumstance. ***(Avoidance)*** |  |
|  |  |  |  |
| ***Assertiveness*** | It turns out, one of Y's friends tells X that Y likes him/her. Protagonist question: Knowing this, what do you think X would do? | Go up and speak with Y directly. ***(High)*** | 0: No 1: Low 2: Moderate 3: High |
|  |  | Do nothing and hope Y will notice X. ***(No)*** |  |
|  |  | Give Y "a look". ***(Low)*** |  |
|  |  | Walk by and bump into Y "accidentally". ***(Moderate)*** |  |
|  |  |  |  |
|  |  | Ignore Y. ***(No)*** |  |
| ***Peer Understanding towards Gender Stigma*** | X has always felt like playing with peers from the opposite sex. One day after school X approached such group of peers and asked to join their play but got denied. Protagonist question: What do most of X's same-sex classmates think about him/her not being allowed to join? | They think X should be able to participate. ***(High)*** | 0: Low 1: High |
|  |  |  |  |
|  |  | They think it is unfair, but they are never allowed to play with peers with opposite sex. ***(High)*** |  |
|  |  |  |  |
|  |  |  |  |
|  |  | They think X is weird and just makes trouble for himself/herself. ***(Low)*** |  |
|  |  |  |  |
|  |  | They think X is probably homosexual. ***(Low)*** |  |
| ***Social Inclusion*** | Same vignette but asks different question: What do you think that group of peers would do when X asks to join in? | They welcome X to join, just like anyone else. ***(Yes)*** | 0: No 1: Some 2: Yes |
|  |  |  |  |
|  |  | They say that X can't join since the conversation is for opposite sex only. ***(No)*** |  |
|  |  |  |  |
|  |  | They refuse and call him names for wanting to act like from opposite sex. ***(No)*** |  |
|  |  |  |  |
|  |  | They allow X to join because X is good at the games they play. ***(Yes)*** |  |
|  |  |  |  |
|  |  | They allow X to join, but tease X. ***(Some)*** |  |
| ***Emotional Response (Puberty)*** | Recently, X has seen some body changes in himself/herself, of which changes he/she did not have before. Protagonist question: How is X feeling about the body changes and the fact that X is going through puberty? | X is happy that he/she is becoming a grown-up. ***(Positive)*** | 0: Very negative 1: Negative 2: Neutral 3: Positive |
|  |  |  |  |
|  |  | X thinks that he/she is sick and something is terribly wrong. ***(Very negative)*** |  |
|  |  |  |  |
|  |  | X is embarrassed about the changes he/she is experiencing. ***(Negative)*** |  |
|  |  |  |  |
|  |  | X is worried about the changes. ***(Negative)*** |  |
|  |  | X is really sad about becoming an adult. ***(Very negative)*** |  |
|  |  |  |  |
|  |  | X is confused and wants more information about the changes he/she is experiencing. ***(Neutral)*** |  |
|  |  |  |  |
|  |  |  |  |
| ***Proactiveness (Puberty)*** | X is confused about the changes that he/she is experiencing. Protagonist question: What will he/she do next? | Tells no one that he/she has finally started puberty. ***(No action)*** | 0: Negative action 1: No action 2: Positive action |
|  |  |  |  |
|  |  | Speaks with someone and asks for advice. ***(Positive action)*** |  |
|  |  |  |  |
|  |  | Searches for information about body changes. ***(Positive action)*** |  |
|  |  |  |  |
|  |  | Tries to hide body changes. ***(Negative action)*** |  |
| ***Parent Response (Puberty)*** | X tells his(her) father(mother) about the body changes. Protagonist question: How do parent most likely to first react to the fact that X has finally begun puberty? | His(her) father(mother) is happy and tells X that he/she is becoming a man(woman). ***(Positive)*** | 0: Negative 1: Positive |
|  |  |  |  |
|  |  |  |  |
|  |  | His(her) father(mother) makes fun of him(her). ***(Negative)*** |  |
|  |  |  |  |
|  |  | His(her) father(mother) tells X that he/she is growing up and should take on responsibilities. ***(Positive)*** |  |
|  |  |  |  |
|  |  |  |  |
|  |  | His(her) father(mother) tells X that he/she should no longer play with girls/boys. ***(Negative)*** |  |
|  |  |  |  |
|  |  |  |  |
|  |  | His(her) father(mother) will teach him/her about hygiene and relevant knowledge. ***(Positive)*** |  |
|  |  |  |  |
|  |  |  |  |
| ***Peer Response (Puberty)*** | X has been the subject of jokes and teased by more mature friends. Now his(her) friends start seeing that X is also maturing. Protagonist question: What do you think the friends are most likely to do? | They will make fun of him/her for being slow. ***(Not at all supportive/low supportive)*** | 0: Not at all supportive/low supportive 1: Moderate supportive 2: Supportive |
|  |  |  |  |
|  |  | They will be too embarrassed to say anything. ***(Not at all supportive/low supportive)*** |  |
|  |  |  |  |
|  |  | They will tell him/her that because now he(she) is a man(woman), it is time to get a girlfriend(boyfriend). ***(Supportive)*** |  |
|  |  |  |  |
|  |  |  |  |
|  |  | They will see it as normal and pay no attention to it. ***(Moderate supportive)*** |  |
|  |  |  |  |
| ***Responsibility (Pregnancy)*** | X's girlfriend (Y) recently realized that she is pregnant and told X that he made her pregnant. Respondent question: How do you think you would react if you were ever in X's situation? | Do nothing and hope it will just go away. ***(Deny responsibility)*** | 0: Deny responsibility 1: Accept responsibility |
|  |  |  |  |
|  |  | Accept the pregnancy but refuse any further involvement with Y. ***(Deny responsibility)*** |  |
|  |  |  |  |
|  |  | Accept the pregnancy and would be happy to have a baby with Y. ***(Accept responsibility)*** |  |
|  |  |  |  |
|  |  | Terminate pregnancy. ***(Deny responsibility)*** |  |
| ***Parental Support (Pregnancy)*** | X's parents know from X's siblings that X's girlfriend is pregnant. Protagonist question: How will X's parents react when they find out the fact? | Kick X out of house. ***(Not supportive)*** | 0: Not supportive 1: Supportive |
|  |  | Say that they will find money for Y to have an abortion. ***(Not supportive)*** |  |
|  |  |  |  |
|  |  | Say they will force X to marry Y as soon as possible. ***(Supportive)*** |  |
|  |  |  |  |
|  |  | Say they will take care of baby no matter what X decided to do with Y. ***(Supportive)*** |  |
|  |  |  |  |
| ***Peer Responsibility (Pregnancy)*** | Same vignette but asked: What do you think your friends would do if they ever were in this kind of situation? | (Encourage girlfriend to) Continue the pregnancy and keep the baby. ***(Accept)*** | 0: Deny responsibility 1: Accept responsibility |
|  |  |  |  |
|  |  | Accept pregnancy but end the relationship. ***(Deny)*** |  |
|  |  |  |  |
|  |  | Get some money for abortion. ***(Deny)*** |  |
|  |  | Accompany girlfriend (or ask boyfriend's accompany) to have an abortion. ***(Deny)*** |  |
|  |  |  |  |
|  |  |  |  |
| X, Y = faked name for boy protagonists or girl protagonists. | |  |  |
