# Supplementary material for: Measuring young adolescent perceptions of relationships: A vignette-based approach to exploring gender equality
Source: PLoS One. 2019 Jun 27;14(6):e0218863. doi: 10.1371/journal.pone.0218863 (PMC6597075; doi:10.1371/journal.pone.0218863)
Supplement: S2 Table — (DOCX) [file pone.0218863.s002.docx]

***S2 Table: Demographic Characteristics for Analytical Sample by Site***

|  | **Kinshasa (N=2586)** | **Shanghai (N=1645)** | **Cuenca**  **(N=484)** |
| --- | --- | --- | --- |
| **Age, *n (%)*** |  |  |  |
| 10 | 528 (20.42) | 12 (0.73) | 93 (19.21) |
| 11 | 543 (21.00) | 275 (16.72) | 104 (21.49) |
| 12 | 568 (21.96) | 533 (32.40) | 102 (21.07) |
| 13 | 503 (19.45) | 589 (35.81) | 102 (21.07) |
| 14 | 444 (17.17) | 236 (14.35) | 83 (17.15) |
| **Sex, *n (%)*** |  |  |  |
| Boy | 1274 (49.27) | 830 (50.46) | 258 (53.31) |
| Girl | 1312 (50.73) | 815 (49.54) | 226 (46.69) |
| **School Enrollment at Interview, *n (%)*** |  |  |  |
| Out-of-school | 718 (27.76) | 0 | 0 |
| In-school | 1868 (72.24) | 1645 (100) | 484 (100) |
| **Education attainment, *n (%)*** |  |  |  |
| Lower than age expected grade | 938 (36.27) | 308 (18.72) | 2 (0.41) |
| Age expected grade or higher | 1648 (63.73) | 1337 (81.28) | 482 (99.59) |
| **Sibling Structure, *n (%)*** |  |  |  |
| No siblings | 56 (2.17) | 1001 (60.85) | 32 (6.61) |
| Same sex siblings only | 245 (9.47) | 147 (8.94) | 126 (26.03) |
| Different sex or mixed sex siblings | 2279 (88.13) | 432 (26.26) | 325 (67.15) |
| *Missing* | 6 (0.23) | 65 (3.95) | 1 (0.21) |
| **Pubertal Onset, *n (%)*** |  |  |  |
| Pre-pubertal | 932 (36.04) | 135 (8.21) | 59 (12.19) |
| Pubertal | 1622 (62.72) | 1344 (81.70) | 404 (83.47) |
| *Missing* | 32 (1.24) | 166 (10.09) | 21 (4.34) |
| **Peer Structure, *n (%)*** |  |  |  |
| No Friends | 107 (4.14) | 83 (5.05) | 12 (2.48) |
| 1-2 friends | 785 (30.36) | 322 (19.57) | 77 (15.91) |
| >2 friends | 1386 (53.60) | 961 (58.42) | 314 (64.88) |
| *Missing* | 308 (11.91) | 279 (16.96) | 81 (16.74) |
| **Frequency of Peer Socialization, *n (%)*** |  |  |  |
| No friends | 107 (4.14) | 83 (5.05) | 12 (2.48) |
| No time spent with friends | 48 (1.86) | 559 (33.98) | 169 (34.92) |
| Often (1-4 times a week) | 1127 (43.58) | 852 (51.79) | 215 (44.42) |
| Nearly every day | 1303 (50.39) | 107 (6.5) | 85 (17.56) |
| *Missing* | 1 (0.04) | 44 (2.67) | 3 (0.62) |
