# Supplementary material for: Measuring young adolescent perceptions of relationships: A vignette-based approach to exploring gender equality
Source: PLoS One. 2019 Jun 27;14(6):e0218863. doi: 10.1371/journal.pone.0218863 (PMC6597075; doi:10.1371/journal.pone.0218863)
Supplement: S1 Text — (DOCX) [file pone.0218863.s003.docx]

**S1 Text.**

**Global Early Adolescent Study**

**
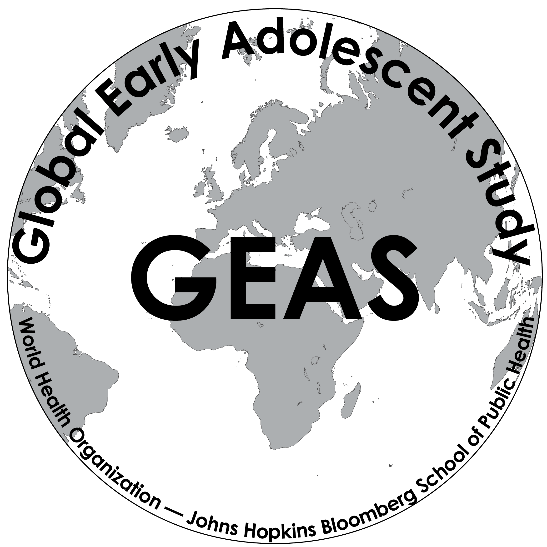
**

**VIGNETTES-BASED MEASURE OF GENDER EQUALITY**

English Version

*This instrument was developed by researchers at the Johns Hopkins Bloomberg School of Public Health with the World Health Organization and partners at research institutions in 15 countries around the world. It is intended for use among 10- to 14-year-old adolescents living in poor urban or peri-urban areas.*

*For more information on this tool or the GEAS, contact Principal Investigator Robert Blum at* [*rblum@jhu.edu*](mailto:rblum@jhu.edu)*.*

Contents

FEMALE VERSION 3

1. Protagonist (P) Likes Antagonist (A) 3

2. Teasing/Bullying Adolescents Preferring to Play with the Opposite Sex 4

3. Puberty 6

4. Pregnancy 8

MALE VERSION 10

1. Protagonist (P) Likes Antagonist (A) 10

2. Teasing/Bullying Adolescents Preferring to Play with the Opposite Sex 11

3. Puberty 13

4. Pregnancy 15

# FEMALE VERSION

## 1. Protagonist (P) Likes Antagonist (A)

***P is in your grade. She is attracted to A, who is in the same grade, but she doesn’t know him and has never spoken with him. Most of her friends say they have boyfriends but she has never had one before. She wants to get his attention, but is not sure how.***

1. What do you think she would do to get his attention? Pick the best option among the following:

- Ask a friend to tell A that she likes him
- Pass A a note
- Go up and talk to A directly
- Nothing, just wait hoping she will meet A
- Refuse to answer

2. What about you? What option would you choose in that situation? Pick the best option among the following:

- Ask a friend to tell A that you like him
- Pass A a note
- Go up and talk to A directly
- Nothing, just wait hoping you will meet A
- Refuse to answer

3. If the situation was reversed and A was attracted to P, but he didn’t know her, what do you think he would do to get her attention? Pick the best option among the following:

- Ask a friend to tell P that he likes her
- Pass P a note
- Go up and talk to P directly
- Nothing, just wait hoping he will meet P
- Refuse to answer

***It is Friday, and P and A are both at the party [site-specific] with their friends. P (who is attracted to A) sees that A is standing in a corner across the room.***

4. What would it take for P to talk to him? Pick the best option among the following:

- Her friends challenged or encouraged her to go up to A
- A was alone
- A came up to her or noticed her in another way
- She knew from a friend that A liked her
- She would not talk to A under any circumstance
- Refuse to answer

5. As it turns out, one of A’s friends tells P that A likes her. Knowing this, what do you think she would do? Pick the best option among the following:

- Go up and speak with A directly
- Do nothing and hope A will notice her
- Give A “a look”
- Walk by and bump into A “accidentally”
- Ignore A
- Refuse to answer

6. Now think about a situation at the party where the roles are reversed. A is attracted to P and sees her in the corner of the room. He doesn’t know what she thinks of him. What would it take for A to talk to P? Pick the best option among the following:

- His friends challenged or encouraged him to go up to P
- P was alone
- P came up to him or noticed him in another way
- He knew from a friend that P liked him
- He would not talk to P under any circumstance
- Refuse to answer

7. As it turns out, one of P’s friends tells A that P likes him. Knowing this, what do you think he would do? Pick the best option among the following:

- Go up and speak with P directly
- Do nothing and hope P will notice him
- Give P “a look”
- Walk by and bump into P “accidentally”
- Ignore P
- Refuse to answer

## 2. Teasing/Bullying Adolescents Preferring to Play with the Opposite Sex

***Ever since she was a child, P has always felt bored playing with other girls, and prefers to play with boys. She is now 13 and one day after school she sees her male classmates standing in a circle chatting in the middle of the playground. P wants to join them. She approaches them asking to join in.***

1. What do you think the boys would do when P asks to join in? Pick the best option among the following:

- They welcome her to join, just like anyone else
- They say that she can’t join since the conversation is for boys only
- They refuse and call her names for wanting to act like a boy
- They allow her to join because she is good at the games they play
- They allow her to join, but tease her
- Refuse to answer

***The boys refuse to allow P to join their conversation.***

2. Why do you think they refuse to let her join them? Pick the best option among the following:

- Because they feel uncomfortable discussing boy stuff with a girl
- Because they think girls should hang out with girls and boys with boys
- Because they think that P is very weird/unusual
- Because they don’t want to be friends with someone they think is a lesbian/homosexual
- Refuse to answer

***Conversation turns to sports and the boys start preparing for a soccer [site-specific: popular local game that boys play] game. Again, P asks to join. Again, she is told no.***

3. What do most of P’s female classmates think about her not being allowed to join the boys’ soccer game? Pick the best option among the following:

- They think she should be able to participate
- They think it is unfair, but girls are never allowed to play with boys
- They think P is weird and just makes trouble for herself
- They think she is probably lesbian/homosexual
- Refuse to answer

***Imagine that the situation was reversed and it was a boy, A, who preferred to play with girls and who saw his female classmates standing in a circle talking and wanted to join them.***

***A approaches them, asking to join in.***

4. What do you think the girls would do when A asks to join in? Pick the one best option among the following:

- They welcome him to join, just like anyone else
- They say that he can’t join since the conversation is for girls only
- They refuse and tease him for wanting to act like a girl
- They allow him to join because he is good at the games they play
- They allow him to join, but tease him
- Refuse to answer

***The girls refuse to allow A to join their conversation.***

5. Why do you think they refuse to let A join them? Pick the best option among the following:

- Because they feel uncomfortable discussing girl stuff with a boy
- Because they think girls should hang out with girls and boys with boys
- Because they think that A is very weird/unusual
- Because they don’t want to be friends with someone they think is gay/homosexual
- Refuse to answer

6. If you were with a group of female friends and a boy like A asked to join, would you let him join? Pick the best option among the following:

- Yes, I would have let him join like anyone else
- Yes, I would have let him join, but I would not want to have anything to do with him
- No, because a guy like that would have made trouble for the group
- No, because a guy like that is weird or gay and I would rather not have him around
- No, because he would have been bullied if he had joined and it would not have been fun for the rest of us
- No, because I would not want to confront my female friends who are opposed
- Refuse to answer

## 3. Puberty

***P is 15 years old. She has been worried for a long time that all the other girls in her group were becoming curvier and starting to develop breasts. Until recently, P had seen none of those changes herself. The other day she got her first period and she has started noticing hair where she didn’t have it before.***

1. How is P feeling about the body changes she is experiencing, and the fact that she is going through puberty? Pick the best option among the following:

- She is happy that she is becoming a grown-up
- She thinks that she is sick and something is terribly wrong
- She is embarrassed about the changes she is experiencing
- She is worried about the changes
- She is really sad about becoming an adult
- She is confused and wants more information about the changes she is experiencing
- Refuse to answer

***P is confused about the changes that she is experiencing.***

2. What will she do next? Pick the best option among the following:

- Tell no one that she has finally started puberty
- Speak with someone and ask for advice
- Search for information without talking to anyone about having her period
- Try to hide her body changes
- Refuse to answer

***P tells her mother about her body changes.***

3. How is her mother most likely to **first** react to the fact that P has finally begun puberty? Pick the best option among the following:

- Her mother tells P she is happy now that she is becoming a woman
- Her mother makes fun of her
- Her mother tells her that now that she is growing up, it is time to take on more responsibilities at home
- Her mother tells her she should no longer play with boys
- Her mother will teach her about hygiene and about the meaning of periods
- Refuse to answer

***For a long time, P has been the subject of jokes and teasing by her more mature girl friends. Now her friends start seeing that P is also maturing.***

4. What do you think they are most likely to do? Pick the best option among the following:

- They will make fun of her for being slow
- They will be too embarrassed to say anything
- They will tell her that because now that she is a woman, it is time to get a boyfriend
- They will see it as normal and pay no attention to it
- Refuse to answer

## 4. Pregnancy

***P is 15 years old and in 9^th^ grade [or appropriate grade per site]. Her boyfriend, A, is also 15 years old. Recently, P realized that she is pregnant and told A that he had made her pregnant. The next day, P’s best friend notices that she is not herself and asks her what the problem is.***

1. How do you think P is **feeling**? Pick the best option among the following:

- Confused
- Scared
- Happy
- Proud
- Angry
- Sad
- Refuse to answer

2. How do you think P will react when she realizes she is pregnant? Pick the best option among the following:

- Do nothing and hope it will just go away
- Accept the pregnancy but refuse any further involvement with A
- Accept the pregnancy since she is happy to have a baby with A
- Refuse to carry the pregnancy and try to stop it somehow
- Refuse to answer

3. How do you think **you** would react if you were in P’s situation? Pick the best option among the following:

- Do nothing and hope it will just go away
- Accept the pregnancy but refuse any further involvement with A
- Accept the pregnancy since I would be happy to have a baby with A
- Refuse to carry the pregnancy and try to stop it somehow
- Refuse to answer

***P tells her friend that she is pregnant and that A has caused the pregnancy.***

4. What does P’s friend advise her to do? Pick the best option among the following:

- Run away from home
- Have the baby and raise it
- Have the baby and give it up for adoption
- Talk with A and make a decision together
- Get an abortion
- Do whatever A decides
- Refuse to answer

***P’s younger sister finds out from P’s friend that she is pregnant and tells their parents.***

5. How will P’s parents react when they find out that their daughter is pregnant? Pick the best option among the following:

- Kick P out of the house
- Say that they will find the money for A to have an abortion
- Say they will force P to marry A as soon as possible
- Say they will take care of the baby no matter what P decides to do with A
- Refuse to answer

6. What do **you** think P **should** do with her pregnancy? Pick the best option among the following:

- Continue the pregnancy and keep the baby
- End her relationship with A and continue the pregnancy alone
- Ask A for help getting money for an abortion, but deal with it alone
- Ask A to accompany her to have the abortion
- Refuse to answer

7. What do you think **your friends** would do if they ever were in this kind of situation? Pick the best option among the following:

- Continue the pregnancy and keep the baby
- End the relationship with A and continue the pregnancy alone
- Ask A for help getting money for an abortion, but deal with it alone
- Ask A to accompany them to have the abortion
- Refuse to answer

# MALE VERSION

## 1. Protagonist (P) Likes Antagonist (A)

***P is in your grade. He is attracted to A, who is in the same grade, but he doesn’t know her and has never spoken with her. Most of his friends say they have girlfriends but he has never had one before. He wants to get her attention, but is not sure how.***

1. What do you think he would do to get her attention? Pick the best option among the following:

- Ask a friend to tell A that he likes her
- Pass A a note
- Go up and talk to A directly
- Nothing, just wait hoping he will meet A
- Refuse to answer

2. What about you? What option would you choose in that situation? Pick the best option among the following:

- Ask a friend to tell A that you like her
- Pass A a note
- Go up and talk to A directly
- Nothing, just wait hoping you will meet A
- Refuse to answer

3. If the situation was reversed and A was attracted to P, but she didn’t know him, what do you think she would do to get his attention? Pick the best option among the following:

- Ask a friend to tell P that she likes him
- Pass P a note
- Go up and talk to P directly
- Nothing, just wait hoping she will meet P
- Refuse to answer

***It is Friday, and P and A are both at a party [site-specific] with their friends. P (who is attracted to A) sees that A is standing in a corner across the room.***

4. What would it take for P to talk to her? Pick the best option among the following:

- His friends challenged or encouraged him to go up to A
- A was alone
- A came up to him or noticed him in another way
- He knew from a friend that A liked him
- He would not talk to A under any circumstance
- Refuse to answer

5. As it turns out, one of A’s friends tells P that A likes him. Knowing this, what do you think he would do? Pick the best option among the following:

- Go up and speak with A directly
- Do nothing and hope A will notice him
- Give A “a look”
- Walk by and bump into A “accidentally”
- Ignore A
- Refuse to answer

6. Now think about a situation at the party where the roles are reversed. A is attracted to P and sees him in the corner of the room. She doesn’t know what he thinks of her. What would it take for A to talk to P? Pick the best option among the following:

- Her friends challenged or encouraged her to go up to P
- P was alone
- P came up to her or noticed her in another way
- She knew from a friend that P liked her
- She would not talk to P under any circumstance
- Refuse to answer

7. As it turns out, one of P’s friends tells A that P likes her. Knowing this, what do you think she would do? Pick the best option among the following:

- Go up and speak with P directly
- Do nothing and hope P will notice her
- Give P “a look”
- Walk by and bump into P “accidentally”
- Ignore P
- Refuse to answer

## 2. Teasing/Bullying Adolescents Preferring to Play with the Opposite Sex

***Ever since he was a child, P has always felt bored playing with other boys, and prefers to play with girls. He is now 13 and one day after school he sees his female classmates standing in a circle chatting in the middle of the playground. P wants to join them. He approaches them asking to join in.***

1. What do you think the girls would do when P asks to join in? Pick the best option among the following:

- They welcome him to join, just like anyone else
- They say that he can’t join since the conversation is for girls only
- They refuse and call him names for wanting to act like a girl
- They allow him to join because he is good at the games they play
- They allow him to join, but tease him
- Refuse to answer

***The girls refuse to allow P to join their conversation.***

2. Why do you think they refuse to let him join them? Pick the best option among the following:

- Because they feel uncomfortable discussing girl stuff with a boy
- Because they think boys should hang out with boys and girls with girls
- Because they think that P is very weird/unusual
- Because they don’t want to be friends with someone they think is a gay/homosexual
- Refuse to answer

***Conversation turns to games and the girls start playing jump rope together [site-specific: popular local activity that girls engage in]. Again, P asks to join. Again, he is told no.***

3. What do most of P’s male classmates think about him not being allowed to join jump rope with the girls? Pick the best option among the following:

- They think he should be able to participate
- They think it is unfair, but boys are never allowed to play with girls
- They think P is weird and just makes trouble for himself
- They think he is probably gay/homosexual
- Refuse to answer

***Imagine that the situation was reversed and it was a girl, A, who preferred to play with boys and who saw her male classmates standing in a circle talking and wanted to join them.***

***A approaches them, asking to join in.***

4. What do you think the boys would do when A asks to join in? Pick the one best option among the following:

- They welcome her to join, just like anyone else
- They say that she can’t join since the conversation is for boys only
- They refuse and tease her for wanting to act like a boy
- They allow her to join because she is good at the games they play
- They allow her to join, but tease her
- Refuse to answer

***The boys refuse to allow A to join their conversation.***

5. Why do you think they refuse to let A join them? Pick the best option among the following:

- Because they feel uncomfortable discussing boy stuff with a girl
- Because they think boys should hang out with boys and girls with girls
- Because they think that A is very weird/unusual
- Because they don’t want to be friends with someone they think is lesbian/homosexual
- Refuse to answer

6. If you were with a group of male friends and a girl like A asked to join, would you let her join? Pick the best option among the following:

- Yes, I would have let her join like anyone else
- Yes, I would have let her join, but I would not want to have anything to do with her
- No, because a girl like that would have made trouble for the group
- No, because a girl like that is weird or a lesbian and I would rather not have her around
- No, because she would have been bullied if she had joined and it would not have been fun for the rest of us
- No, because I would not want to confront my male friends who are opposed
- Refuse to answer

## 3. Puberty

***P is 15 years old. He has been worried for a long time that all the other boys in his group were becoming taller and also had some facial hair. Until recently, P had seen none of those changes himself. The other evening he woke up and his underwear was wet because he had had a “wet dream,” and he has started noticing hair where he didn’t have it before.***

1. How is P feeling about the body changes he is experiencing, and the fact that he is going through puberty? Pick the best option among the following:

- He is happy that he is becoming a grown-up
- He thinks that he is sick and something is terribly wrong
- He is embarrassed about the changes he is experiencing
- He is worried about the changes
- He is really sad about becoming an adult
- He is confused and wants more information about the changes he is experiencing
- Refuse to answer

***P is confused about the changes that he is experiencing.***

2. What will he do next? Pick the best option among the following:

- Tell no one that he has finally started puberty
- Speak with someone and ask for advice
- Search for information without talking to anyone about the wet stains on his underwear
- Try to hide his body changes
- Refuse to answer

***P tells his father about his body changes.***

3. How is his father most likely to **first** react to the fact that P has finally begun puberty? Pick the best option among the following:

- His father tells P he is happy now that he is becoming a man
- His father makes fun of him
- His father tells him that now that he is growing up, it is time to take on more responsibilities at home
- His father tells him he should no longer play with girls
- His father will teach him about hygiene and about the meaning of nocturnal emission/wet dreams
- Refuse to answer

***For a long time, P has been the subject of jokes and teasing by his more mature guy friends. Now his friends start seeing that P is also maturing.***

4. What do you think the friends are most likely to do? Pick the best option among the following:

- They will make fun of him for being slow
- They will be too embarrassed to say anything
- They will tell him that because now that he is a man, it is time to get a girlfriend
- They will see it as normal and pay no attention to it
- Refuse to answer

## 4. Pregnancy

***P is 15 years old and in 9^th^ grade [or appropriate grade per site]. His girlfriend, A, is also 15 years old. Recently, A realized that she is pregnant and told A that he had made her pregnant. The next day, P’s best friend notices that he is not himself and asks him what the problem is.***

1. How do you think P is **feeling**? Pick the best option among the following:

- Confused
- Scared
- Happy
- Proud
- Angry
- Sad
- Refuse to answer

2. How do you think P will react when he realizes that A is pregnant? Pick the best option among the following:

- Do nothing and hope it will just go away
- Accept the pregnancy but refuse any further involvement with A
- Accept the pregnancy since he is happy to have a baby with A
- Tell A not to continue the pregnancy and try to stop it somehow
- Refuse to answer

3. How do you think **you** would react if you were in P’s situation? Pick the best option among the following:

- Do nothing and hope it will just go away
- Accept the pregnancy but refuse any further involvement with A
- Accept the pregnancy since I would be happy to have a baby with A
- Tell A not to continue the pregnancy and try to stop it somehow
- Refuse to answer

***P tells his friend that A is pregnant and that he has caused the pregnancy.***

4. What does P’s friend advise him to do? Pick the best option among the following:

- Run away from home
- Encourage A to have the baby and raise it
- Encourage A to have the baby and give it up for adoption
- Talk with A and make a decision together
- Encourage A to get an abortion
- Do whatever A decides
- Refuse to answer

***P’s younger brother finds out from P’s friend that P’s girlfriend A is pregnant and tells their parents.***

5. How will P’s parents react when they find out that their son's girlfriend is pregnant? Pick the best option among the following:

- Kick P out of the house
- Say that they will find the money for A to have an abortion
- Say they will force P to marry A as soon as possible
- Say they will take care of the baby no matter what P decides to do with A
- Refuse to answer

6. What do **you** think P **should** do about A’s pregnancy? Pick the best option among the following:

- Encourage A to continue the pregnancy and keep the baby
- End his relationship with A even if she decides to continue the pregnancy alone
- Help A get money for an abortion, but leave her to deal with it alone
- Accompany A to have the abortion
- Refuse to answer

7. What do you think **your friends** would do if they ever were in this kind of situation? Pick the best option among the following:

- Encourage A to continue the pregnancy and keep the baby
- End the relationship with A even if she decides to continue the pregnancy alone
- Help A get money for an abortion, but leave her to deal with it alone
- Accompany A to have the abortion
- Refuse to answer
