# Supplementary material for: Measuring young adolescent perceptions of relationships: A vignette-based approach to exploring gender equality
Source: PLoS One. 2019 Jun 27;14(6):e0218863. doi: 10.1371/journal.pone.0218863 (PMC6597075; doi:10.1371/journal.pone.0218863)
Supplement: S2 Text — (DOCX) [file pone.0218863.s004.docx]

**S2 Text. Dropped Vignette Measures during Vignettes Development**

# FEMALE VERSION

## 1. Protagonist (P) Wants to Go to Movies with Friends

***One day after school, P and a few of her classmates (mixed boys and girls) decide to see a movie. The film will not end until 9 o’clock at night. Since she will get home late, she asks her mother’s permission to go to the movie.***

1. What do you think she will tell her mother in order to get her permission to go to the movie?

- She tells her mother that she is going with female and male friends
- She tells her mother that she is going only with female friends
- She tells her mother that she is going to her classmate’s home to study
- She goes to the movie without telling her mother anything at all
- Other
- I refuse to answer

2. What would you tell your mother in order to get her permission to go out with friends?

- You would tell her that you are going with female and male friends
- You would tell her that you are going only with female friends
- You would tell her that you are going to the movies without telling her who you are going with
- You would tell her that you are going to a classmate’s home to study for the evening
- You would go to the movie without telling your mother anything at all
- I refuse to answer

3. P tells her mother that she wants to see a movie and that she is going with her female and male friends. What do you think her mother will do?

- Refuse to let P go
- Agree to let P go to the movie, if she or her older brother goes with her
- Agree to let P go to the movie, without any condition
- Tell her to speak with her father. She will agree if he does
- I refuse to answer

***P’s mother refuses to let her go to the movie. P tries again and explains to her mother exactly with whom she is going, what film they will see, and guarantees to be back at a certain time. Her mother still does not agree to let her go.***

4. Why do you think P’s mother refuses to let her go to the movie? Select the most important reason.

- She is worried about P’s safety coming home after the movie by herself
- She is worried that P’s friends may be a bad influence
- She is worried that P will start going out with boys
- She is worried that the movie may not be suitable for young girls
- She is worried that going out will affect her schoolwork and grades
- She is worried about P’s father’s reaction
- I refuse to answer

5. How do you think P feels when her mother refuses to let her go to the movie?

- She feels embarrassed to tell her friends
- She feels angry at her mother
- She understands and respects her mother’s decision
- She feels sad/upset that her mother continues to treat her like a child
- She worries about what she is going to tell her friends
- I refuse to answer

6. What do you think P will do when her mother refuses to let her go to the movie?

- Obey her
- Beg her, hoping that she will change her mind
- Argue with her
- Try to get her father’s permission
- Ignore her mother and go to the movie anyway
- I refuse to answer

7. What would you do in P’s situation?

- Obey your mother
- Continue to beg your mother, hoping that she will change her mind
- Argue with your mother, saying that she does not understand what it is like to be a teenager today
- Try to get your father’s permission
- Ignore your mother and go to the movie anyway
- I refuse to answer

## 2. Deal with Appropriate Clothing

***P has a favorite dress that she got one year ago. Lately her body has started to change – she is taller and has become more “curvy” as her breasts and hips are growing. The dress is now very short and tight on her. One day P’s mother sees P leaving the house in the dress and says that now P is becoming a woman, she shouldn’t be seen in public ‘looking like that’.***

1. What do you think P will do?

- Ask her mother to explain what she means
- Change her clothes with no plans to wear the dress again
- Change her clothes but wear the dress when her mother is not around
- Argue with her mother
- Ignore her mother
- Tell her mother to buy her other clothes
- I refuse to answer

***P hears what her mother is saying, but she does not think that there is a problem with her dress and the way she looks.***

2. What would it take for her to give up her favorite dress?

- If she can get a new dress
- If her female friends tease her
- If a boy she likes says something
- If her father is angry
- If she is called names at school or on the street
- I refuse to answer

***P does not argue with her mother. She changes her dress, but takes it with her and when she is out of the house she changes back. Her mother finds out.***

3. What is P’s mother most likely to say when she finds out that P still wore the dress?

- “This is an embarrassment.”
- “Do you really want girls to get the wrong impression of you?”
- “Do you really want boys to be looking at you?”
- Nothing, her mother doesn’t really care
- I refuse to answer

4. What do you think P’s mother is most likely to do?

- She explains again and gives P one more chance
- She takes the dress away from P
- She punishes P by slapping, spanking or beating her
- She tells P’s father about the situation
- I refuse to answer

# MALE VERSION

## 1. Protagonist (P) Wants to Go to Movies with Friends

***One day after school, P and a few of his classmates (mixed boys and girls) decide to see a movie. The film will not end until 9 o’clock at night. Since he will get home late, he asks his mother’s permission to go to the movie.***

1. What do you think he will tell his mother in order to get her permission to go to the movie?

- He tells his mother that he is going with male and female friends
- He tells his mother that he is going only with male friends
- He tells his mother that he is going to his classmate’s home to study
- He goes to the movie without telling his mother anything at all
- Other
- I refuse to answer

2. What would you tell your mother in order to get her permission to go out with friends?

- You would tell her that you are going with male and female friends
- You would tell her that you are going only with male friends
- You would tell her that you are going to the movies without telling her who you are going with
- You would tell her that you are going to a classmate’s home to study for the evening
- You would go to the movie without telling your mother anything at all
- I refuse to answer

3. P tells his mother that he wants to see a movie and that he is going with his male and female friends. What do you think his mother will do?

- Refuse to let P go
- Agree to let P go to the movie, if she or his older brother goes with him
- Agree to let P go to the movie, without any condition
- Tell him to speak with his father. She will agree if he does
- I refuse to answer

***P’s mother refuses to let him go to the movie. P tries again and explains to his mother exactly with whom he is going, what film they will see, and guarantees to be back at a certain time. His mother still does not agree to let him go.***

4. Why do you think P’s mother refuses to let him go to the movie? Select the most important reason.

- She is worried about P’s safety coming home after the movie by himself
- She is worried that P’s friends may be a bad influence
- She is worried that P will start going out with girls
- She is worried that the movie may not be suitable for young boys
- She is worried that going out will affect his schoolwork and grades
- She is worried about P’s father’s reaction
- I refuse to answer

5. How do you think P feels when his mother refuses to let him go to the movie?

- He feels embarrassed to tell his friends
- He feels angry at his mother
- He understands and respects his mother’s decision
- He feels sad/upset that his mother continues to treat him like a child
- He worries about what he is going to tell his friends
- I refuse to answer

6. What do you think P will do when his mother refuses to let him go to the movie?

- Obey her
- Beg her, hoping that she will change her mind
- Argue with her
- Try to get his father’s permission
- Ignore his mother and go to the movie anyway
- I refuse to answer

7. What would you do in P’s situation?

- Obey your mother
- Beg her
- Argue with her
- Try to get your father’s permission
- Ignore your mother and go to the movie anyway
- I refuse to answer

## 2. Deal with Appropriate Clothing

***P has a favorite pair of shorts that he got one year ago. Lately his body has started to change – he is taller and has become more muscular. The shorts are now very short and tight on him. One day P’s mother sees P leaving the house in the shorts and says that now P is becoming a man, he shouldn’t be seen in public ‘looking like that’.***

1. What do you think P will do?

- Ask his mother to explain what she means
- Change his clothes with no plans to wear the shorts again
- Change his clothes but wear the shorts when his mother is not around
- Argue with his mother
- Ignore his mother
- Tell his mother to buy him other clothes
- I refuse to answer

2. P doesn’t understand… These are his favorite pair of shorts. P argues with his mother and asks why his shorts are inappropriate. What do you think P’s mother says to him?

- “The shorts are too short and tight on you.”
- “Girls will get the wrong idea about you.”
- “The shorts show parts of your body that shouldn’t be shown.”
- “Real men do not wear something like that.”
- “Just do as I say and stop asking so many questions.”
- I refuse to answer

***P does not argue with his mother. He changes his shorts, but takes them with him and when he is out of the house he changes back. His mother finds out.***

3. What is P’s mother most likely to say when she finds out that P still wore the shorts?

- “This is an embarrassment.”
- “Do you really want boys to get the wrong impression of you?”
- “Do you really want girls to be looking at you?”
- Nothing, his mother doesn’t really care
- I refuse to answer

4. What do you think P’s mother is most likely to do?

- She explains again and gives P one more chance
- She takes the shorts away from P
- She punishes P by slapping, spanking or beating him
- She tells P’s father about the situation
- I refuse to answer
