# Supplementary material for: Measuring young adolescent perceptions of relationships: A vignette-based approach to exploring gender equality
Source: PLoS One. 2019 Jun 27;14(6):e0218863. doi: 10.1371/journal.pone.0218863 (PMC6597075; doi:10.1371/journal.pone.0218863)
Supplement: S3 Text — (DOCX) [file pone.0218863.s005.docx]

# S3 Text.

**Vignettes Instrument (Pilot)**

# The Global Early Adolescent Study

VIGNETTE #1: PROTAGONIST (P) LIKES ANTAGONIST (A)

VIGNETTE #2: CAN I GO OUT?

VIGNETTE #3: TEASING/ BULLYING

VIGNETTE #4: PUBERTY

VIGNETTE #5: DRESSING APPROPRIATELY

VIGNETTE #6 #Optional#: PREGNANCY

**MALE VERSION**

VIGNETTE #1: PROTAGONIST (P) LIKES ANTAGONIST (A)

***P is in 7^th^ grade. He is attracted to A, who is in the same grade, but he doesn’t know her and has never spoken with her in person. Most of his friends have girlfriends but he has never had one before. He wants to get her attention, but is not sure how.***

1. **What do you think he is most likely to do?**

- Ask a friend to tell A that P likes her
- Pass her a note
- Tease her
- Go up and talk to her directly
- Nothing, just wait hoping he will meet her
- I refuse to answer

1. **What do you think you would do in that situation?**

- Ask a friend to tell A that you like her
- Pass A a note
- Tease her
- Go up and talk to A directly
- Nothing, just wait hoping you will meet her
- I refuse to answer

***P is confused and does not know what to do. He wants to speak with someone about his feelings/emotions and ask for advice.***

1. **Who do you think he is most likely to turn to for advice?**

- A male friend
- A female friend
- A friend of A
- His sister
- His father
- His mother
- His brother
- His uncle
- No one. He keeps it to himself.
- I refuse to answer

1. **If P decides to approach A directly, what is he most likely to do?**

- Follow A on social media (Facebook/ Instagram/ 2go etc.)
- Wait for A outside of school (along the road) to talk with her
- Hand A a note directly
- Call her on the phone after getting her number from her friend ***(NOTE: can be modified to be texting him/contacting him by other social media)***
- I refuse to answer

***P decides to talk to A himself. He is walking out of school with his friends when they run into a group of girls that includes A. They all start talking about a friend’s party that is happening next Friday. P wants to know if A is going, but is afraid to ask. He feels uncomfortable about it. (NOTE: can be modified to be site‐specific event. What is important is that they have an event where boys and girls will get together.)***

1. **What do you think he is most likely to do?**

- Ask if any of the girls are going to the party
- Ask A directly if she is going to the party
- Get a friend to ask A if she is going to the party
- Say nothing and hope that someone else will ask A if she is going
- Ask A’s friend if she knows if A is going to the party
- I refuse to answer

***It is Friday, and P and A are both at the party with their friends. P sees that A is standing in a corner across the room. What would it take for him to talk to her?***

1. **He would talk to her if:**

- His friends challenged or encouraged him to go up to A
- A was alone
- A came up to him
- A gave him a long look or noticed him in another way
- He knew from a friend that A liked him
- He would not talk to A no matter what
- I refuse to answer

1. **In P’s situation, would you talk to A if…**

- Your friends challenged or encouraged you to go up to A
- A was alone
- A came up to you
- A gave you a long look or noticed you in another way
- You knew from a friend that A liked you
- You would not talk to A no matter what
- I refuse to answer

***It is also quite possible that P might not talk with A at all.***

1. **What is the most likely reason that he might not talk to her? He would not talk to her because…**

- He is too embarrassed/shy
- He is afraid of rejection
- He thinks that she might already have a boyfriend
- He is not allowed to have a girlfriend
- I can’t think of a reason why P would not talk to A
- I refuse to answer

***Actually, P and A meet through a mutual friend. P and A talk and dance several times during the evening. It is now the end of the party.***

1. **What do you think P really wants to happen next?**

- P wants A to be his girlfriend, but wants her to ask him
- P wants to ask A to be his girlfriend, and will ask her directly
- P wants to exchange phone numbers so that they can talk/text later
- P wants to kiss A
- P doesn’t really know what he wants
- I refuse to answer

***P and A exchanged phone numbers and P is now back home and thinks back on his evening.***

1. **How is he feeling? (pick the one that best describes his feeling)**

- Happy
- Proud
- Nervous
- Unsure
- Afraid
- Turned on/excited/ “freaky”/aroused (Note: use local term that connotes arousal)
- I refuse to answer

***He texts his best friend about the evening. What do you think he writes to his friend?***

1. ***________________________________________________________________________***

***________________________________________________________________________***

1. **How do you think P’s *mother* would react if she found out that her son spent the evening with a girl?**

- She would be angry
- She would be happy
- She would be worried
- She would not care
- I refuse to answer

1. **What would P’s *mother* do?**

- She would not allow P to see A
- She would threaten to punish P
- She would encourage P to see A again
- She would not do anything
- I refuse to answer

1. **How do you think P’s *father* would react if he found out that his son spent the evening with a girl?**

- He would be angry
- He would be happy
- He would be worried
- He would not care
- I refuse to answer

1. **What would P’s *father* do?**

- He would not allow P to see A again
- He would threaten or punish P
- He would encourage P to see A again
- He would not do anything
- I refuse to answer

VIGNETTE #2: CAN I GO OUT?

***One day after school, P and a few of his classmates, of whom some are boys and some are girls, decide to see a movie. The film will not end until 9 o’clock at night. Since he will get home late, he asks his mother’s permission to go to the movie. (Note: This is not about going to the movies but rather about going out in a mixed group. Modify both activity and return time as appropriate at your site.)***

1. **What do you think P will tell his mother in order to get her permission to go to the movie?**

- He tells his mother that he is *going with male and female friends*
- He tells his mother that he is *going only with male friends*
- He tells his mother he is going to the movies without telling her who he is going with
- He tells his mother that he is going to his classmate’s home to study
- He goes to the movie without telling his mother anything at all
- I refuse to answer

1. **What would you tell your mother in order to get her permission to go out with friends?**

- You would tell her that you are *going with male and female friends*
- You would tell her that you are *going only with female friends.*
- You would tell her you are going to the movies without telling her who you are going with
- You would tell her that you are going to a classmate’s home to study for the evening
- You would go to the movie without telling your mother anything at all
- I refuse to answer

***P tells his mother that he wants to see a movie and that he is going with his male and female friends.***

1. **What do you think his mother will do?**

- Refuse to let P go without saying why
- Refuse to let P go, and explain why he cannot go
- Refuses to let P go, but promises to take him to see the film another day
- Agree to let P go to the movie, on the condition that she picks P up when the movie is over
- Agree to let P go to the movie, on the condition that she or his older brother goes with him
- Agree to let P go to the movie, without any condition
- Tell him to speak with his father. She will agree if he does.
- I refuse to answer

***P’s mother refuses to let him go to the movie. P tries again and explains to his mother exactly with whom he is going, what film they will see, and guarantees to be back at a certain time. His mother still does not agree to let him go.***

1. **Why do you think P’s mother refuses to let him go to the movie? Select the most important reason.**

- She is worried about P’s safety coming home after the movie by himself
- She is worried that P’s friends may be a bad influence
- She is worried that P will start going out with girls
- She is worried that the movie may not be suitable for young boys
- She is worried that going out will affect his schoolwork and grades
- She is worried about P’s father’s reaction
- I refuse to answer

1. **How do you think P feels when his mother refuses to let him go to the movie?**

- He feels embarrassed to tell his friends
- He feels angry at his mother
- He understands and respects his mother’s decision
- He feels sad/upset that his mother continues to treat him like a child
- He worries about what he is going to tell his friends
- I refuse to answer

1. **What do you think P will do when his mother refuses to let him go to the movie?**

- Obey his mother and not go to the movie
- Continue to beg her, hoping that she will change her mind
- Argue with his mother, saying that she does not understand what it is like to be a teenager today
- Try to get his father’s permission
- Ignore his mother and go to the movie anyway
- I refuse to answer

1. **What would you do in P’s situation?**

- Obey your mother
- Continue to beg your mother, hoping that she will change her mind
- Argue with your mother, saying that she does not understand what it is like to be a teenager today
- Try to get your father’s permission
- Ignore your mother and go to the movie anyway
- I refuse to answer

VIGNETTE #3: TEASING/ BULLYING

***Ever since he was a child, P has always felt bored playing with other boys, and prefers to play with girls. He is now 13 and one day after school, his female classmates are standing in a circle chatting in the middle of the playground. P wants to join them. He approaches them asking to join in.***

1. **What do you think his female classmates do when P asks to join in?**

- They welcome him to join, just like anyone else
- They say that he can’t join since the conversation is for girls only
- They refuse and call him names for wanting to talk about girl stuff
- They allow him to join because he is harmless
- They allow him to join because he is good at sports they play
- They allow him to join, but tease him
- I refuse to answer

***The girls refuse to allow P to join their conversation.***

1. **Why do you think they refuse to let him join their circle?**

- Because they think that boys should not want to talk about girl stuff
- Because they think boys should hang out with boys and girls with girls
- Because they think that P is very weird/unusual
- Because they don’t want to be friends with someone they think is gay/homosexual
- I refuse to answer

***Conversation turns to jump rope (popular local game that girls play). Again, P asks to join. Again he is told no.***

1. **What do most of P’s male classmates think about him not being allowed to make join the girls’ game?**

- They think he should be able to participate
- They think it is unfair, but boys are never allowed to play with girls
- They don’t understand why he even asked
- They think he is weird and just makes trouble for himself
- They think he is probably gay/homosexual
- I refuse to answer

***Since P is not allowed to join the girls, he tries to join the boys’ group instead.***

1. **What do you think the other boys will do?**

- They welcome him, just like anyone else
- They let him join the group, but ignore him
- They say that he can’t join because he is not a real boy
- They allow him to join them but tease him
- I refuse to answer

1. **What are most of P’s male classmates most likely to think of him for wanting to join girls’ conversations and activities?**

- They admire him for acting as he wishes
- They think that P is weird
- They disapprove of P’s behavior
- They think that P is probably gay/homosexual
- I refuse to answer

1. **All the boys and girls refuse to play with P. How do you think he feels?**

- He does not really care
- He is confused and does not understand why he cannot play
- He is angry that nobody likes him for who he is
- He is sad and feels rejected
- He is scared that something is wrong about him
- I refuse to answer

1. **How do you think P’s *mother* reacts to the fact that P behaves more like a girl than a boy?**

- His mother tells P that he needs to start behaving “like a boy”
- His mother encourages P to be who he wants to be
- His mother is embarrassed by P
- His mother worries what the neighbors will think
- His mother doesn’t care about P’s behavior
- His mother takes him to a doctor/local healer/pastor
- I refuse to answer

1. **How do you think P’s *father* reacts to his preference for girl things?**

- His father says that P needs start acting “like a boy”
- His father encourages P to be who he wants to be
- His father is embarrassed by P
- His father worries what the neighbors will think
- His father takes him to a doctor/local healer/pastor
- His father doesn’t care one way or another about P’s behavior
- I refuse to answer

VIGNETTE #4: PUBERTY

***P is 15 years old. He has been worried for a long time that all the other boys in his group were growing taller and that they were starting to develop beards. Until recently P has seen none of those changes himself. The other evening he woke up and his underwear was wet and stained; and he has started noticing hair where he didn’t have it before like under his arms.***

**1. What is P most likely to feel about the body changes that he is experiencing?**

- He is happy that he is becoming a grownup
- He is worried that his friends will tease him
- He thinks that he is sick and something is terribly wrong
- He is embarrassed about the changes he is experiencing
- He is really sad about becoming an adult
- He is confused and wants more information about the changes he is experiencing
- I refuse to answer

***P is confused about the changes that he is experiencing.***

1. **What is he most likely to do next?**

- Tell no one that he has finally started puberty
- Speak with someone about his concerns
- Search for information without talking to anyone
- Try to hide his body changes
- I refuse to answer

1. **He decides to speak with someone. Who is he most likely to speak with?**

- His father
- His mother
- His grandfather  A male friend
- A female friend
- His older brother
- His teacher
- His uncle
- His cousin
- I refuse to answer

***P tells his father about his body changes.***

**4. How is his father most likely to *first* react that he has finally begun puberty?**

- His father tells P he is happy for him
- His father says he is worried about what the changes mean for him
- His father teases him
- His father punishes him
- His father tells him that now that he is growing up, it is time to take on more responsibilities at home
- His father tells him that he now he should no longer play with girls
- His father tells him that he needs to be careful about how he dresses when he goes out of the house
- His father will teach him about hygiene and about the meaning of wet dreams/nocturnal emission
- I refuse to answer

**5. P knows that his father has told his mother that he spoke with him about his changes. What is his mother’s most likely reaction?**

- His mother tells P she is happy for him
- She says she is worried about what the changes mean for him
- His mother teases him
- His mother punishes him
- His mother tells P that now that he is growing up, it is time to take on more responsibilities at home
- His mother tells him that he now needs to be careful around girls
- His mother tells him that he needs to be careful about how he dresses when he goes out of the house
- His mother tells the father to talk to P
- I refuse to answer

***For years, P has been the subject of jokes and teasing by his more mature guy friends. Now they start seeing that P is also maturing.***

**6. What do you think they are most likely to do?**

- They will tell him how excited they are for him
- They will continue to tease him for being slow
- They will be too embarrassed to say anything
- They will tell him it is time to get a girlfriend
- They will tell him what it means to be a man
- I refuse to answer

***P’s friends tease him for being slower than everyone else to enter puberty, and continue to tease him over the next few weeks. He is feeling more and more alone. He decides to speak with his older brother.***

**8. What is his brother most likely to say to him?**

- “You need to find other friends”
- “It is time that you found a girlfriend”
- “You need to stay at home more”
- “You should be proud that you are growing up”
- I refuse to answer

VIGNETTE #5: DRESSING APPROPRIATELY

***P has a favorite pair of shorts that he got one year ago. Lately his body has started to change – he is taller and has become more muscular. The shorts are now very short and tight on him.***

***One day P’s mother sees P leaving the house in the shorts and says that now that P is “becoming a man, he shouldn’t be seen in public ‘looking like that.’”***

1. **What do you think P will do?**

- Ask his mother to explain what she means
- Change his clothes with no plans to wear the shorts again
- Change his clothes but wear the shorts when his mother is not around
- Argue with his mother
- Ignore his mother
- Tell his mother to buy him other clothes
- I refuse to answer

***P doesn’t understand… These are his favorite pair of shorts. P argues with his mother and asks why his shorts are inappropriate.***

1. **What do you think P’s mother says to him?**

- “The shorts are too short and tight on you”
- “Girls will get the wrong idea about you”
- “The shorts show parts of your body that shouldn’t be shown”
- “Real men do not wear something like that”
- “Just do as I say and stop asking so many questions”
- I refuse to answer

***P hears what his mother is saying, but he does not think that there is a problem with his shorts and the way he looks.***

1. **What would it take for him to give up his favorite pair of shorts?**

- If he can get a new pair
- If his male friends tease him
- If a girl he likes says something
- If his father is angry
- If he is called names at school or on the street
- I refuse to answer

***P does not argue with his mother. He changes his shorts, but takes them with him and when he is out of the house he changes back. His mother finds out.***

1. **What is P’s mother most likely to say when she finds out that P still wore the shorts?**

- “This is an embarrassment”
- “Do you really want boys to get the wrong impression of you?”
- “Do you really want girls to be looking at you?”
- Nothing; his mother doesn’t really care
- I refuse to answer

1. **What do you think P’s mother is most likely to do?**

- She explains again and gives P one more chance
- She takes the shorts away from P
- She punishes P by slapping, spanking or beating him
- She tells P’s father about the situation
- I refuse to answer

VIGNETTE #6 #Optional#: PREGNANCY

***P is 14 years old and in 9^th^ grade (or appropriate grade per site). His girlfriend, A, is also 14 years old. Recently, A realized that she is pregnant, and told P that he is responsible for the pregnancy. P can’t believe it. His best friend notices that P is not himself and asks him what the problem is.***

1. **How do you think P is *feeling*?**

- Confused
- Scared
- Happy
- Angry
- Very sad
- I refuse to answer

1. **How do you think P is most likely to react when he realizes that A is pregnant?**

- Be happy that he is having a baby with A
- Deny that A is pregnant
- Deny that he is responsible for the pregnancy
- Accuse A of trapping him into being a father
- Accept that A is pregnant but refuse any further involvement with her
- I refuse to answer

1. **How do you think you would react if you were ever in P’s situation?**

- I would be happy that I was having a baby with A
- Deny that A is pregnant
- Deny that I am responsible for the pregnancy
- I would accuse A of trapping me into being a father
- I would accept that A is pregnant but refuse any further involvement with her
- I refuse to answer

***P tells his friend that his girlfriend A is pregnant and that he has caused the pregnancy.***

1. **What is his friend most likely to advise P to do?**

- Run away from home
- Tell A to have the baby
- Ask A to give the child up for adoption
- “Be a man” and take responsibility
- Ask A to get an abortion
- I refuse to answer

***P is scared and decides to run away from home. He tells his friend of his plan, who in turn tells P’s younger sister. His younger sister then tells their parents that P’s girlfriend is pregnant and P is responsible.***

1. **How will P’s parents react when they find out that their son’s girlfriend is pregnant? They will…**

- Accuse A of tricking P into having sex
- Kick P out of the house
- Say that they will find the money for A to have an abortion
- Say they will force P to marry A as soon as possible
- Say they will take care of the baby no matter what P decides to do with A
- I refuse to answer

***P’s parents insist that their son is not responsible for the pregnancy. They say that P is too young and “innocent” to be having sex, and accuse A of tricking their son into having sex with her.***

1. **What will P do next?**

- He denies responsibility
- He decides to end the relationship with A after the baby is born  He marries A as soon as he can
- He helps A get an abortion
- He runs away from home
- He does nothing and just waits to see what happens next
- I refuse to answer

***P thinks A should end the pregnancy and finds a place where A can have an abortion at a good price.***

1. **What is P most likely to do next?**

- Change his mind at the last minute and ask A to continue the pregnancy/keep the baby
- End his relationship with A and let her continue the pregnancy alone
- Help A get money for an abortion, but leave her to deal with it alone
- Accompany A to have the abortion
- Try to force A to have an abortion, but provide no help
- I refuse to answer

1. **What do *you* think P *should* do in this situation?**

- Ask A to continue the pregnancy/keep the baby
- End his relationship with A and let her continue the pregnancy alone
- Help A get money for an abortion, but leave her to deal with it alone
- Accompany A to have the abortion
- Try to force A to have an abortion, but provide no help
- I refuse to answer

1. **What do you think *your friends would do* if they ever were in this kind of situation?**

- Ask A to continue the pregnancy/keep the baby
- End his relationship with A and let her continue the pregnancy alone
- Help A get money for an abortion, but leave her to deal with it alone
- Accompany A to have the abortion
- Try to force A to have an abortion, but provide no help
- I refuse to answer

**FEMALE VERSION**

VIGNETTE #1: PROTAGONIST (P) LIKES ANTAGONIST (A)

***P is in 7^th^ grade. She is attracted to A, who is in the same grade, but she doesn’t know him and has never spoken with him in person. Most of her friends have boyfriends but she has never had one before. She wants to get his attention, but is not sure how.***

1. **What do you think she is most likely to do?**

- Ask a friend to tell A that P likes him
- Pass him a note
- Tease him
- Go up and talk to him directly
- Nothing, just wait hoping she will meet him
- I refuse to answer

1. **What do you think you would do in that situation?**

- Ask a friend to tell A that you like him
- Pass A a note
- Tease him
- Go up and talk to A directly
- Nothing, just wait hoping you will meet him
- I refuse to answer

***P is confused and does not know what to do. She wants to speak with someone about her feelings/emotions and ask for advice.***

1. **Who do you think she is most likely to turn to for advice?**

- A male friend
- A female friend
- A friend of A
- Her sister
- Her father
- Her mother
- Her brother
- Her aunt
- No one. She keeps it to herself
- I refuse to answer

1. **If P decides to approach A directly, what is she most likely to do?**

- Follow A on social media (Facebook/ Instagram/ 2go etc.)
- Wait for A outside of school (along the road) to talk with him
- Hand A a note directly
- Call him on the phone after getting his number from his friend ***(NOTE: can be modified to be texting him/contacting him by other social media)***
- I refuse to answer

***P decides to talk to A herself. She is walking out of school with her friends when they run into a group of boys that includes A. They all start talking about a friend’s party that is happening next Friday. P wants to know if A is going, but is afraid to ask. She feels uncomfortable about it. (NOTE: can be modified to be site specific event. What is important is that they have an event where boys and girls will get together)***

1. **What do you think she is most likely to do?**

- Ask if any of the boys are going to the party
- Ask A directly if he is going to the party
- Get a friend to ask A if he is going to the party
- Say nothing and hope that someone else will ask A if he is going
- Ask A’s friend if he knows if A is going to the party
- I refuse to answer

***It is Friday, and P and A are both at the party with their friends. P sees that A is standing in a corner across the room. What would it take for her to talk to him?***

1. **She would talk to him if:**

- Her friends challenged or encouraged her to go up to A
- A was alone
- A came up to her
- A gave her a long look or noticed her in another way
- She knew from a friend that A liked her
- She would not talk to A no matter what
- I refuse to answer

1. **In P’s situation, would you talk to A if…**

- Your friends challenged or encouraged you to go up to A
- A was alone
- A came up to you
- A gave you a long look or noticed you in another way
- You knew from a friend that A liked you
- You would not talk to A no matter what
- I refuse to answer

***It is also quite possible that P might not talk with A at all.***

1. **What is the most likely reason that she might not talk to him? She would not talk to him because…**

- She is too embarrassed/shy
- She is afraid of rejection
- She thinks that he might already have a girlfriend
- She is not allowed to have a boyfriend
- I can’t think of a reason why P would not talk to A
- I refuse to answer

***Actually, P and A meet through a mutual friend. P and A talk and dance several times during the evening. It is now the end of the party.***

1. **What do you think P really wants to happen next?**

- P wants A to be her boyfriend, but wants him to ask her
- P wants to ask A to be her boyfriend, and will ask him directly
- P wants to exchange phone numbers so that they can talk/text later
- P wants to kiss A
- P doesn’t really know what she wants
- I refuse to answer

***P and A exchanged phone numbers and P is now back home and thinks back on her evening.***

**10. How is she feeling? (pick the one that best describes her feeling)**

- Happy
- Proud
- Nervous
- Unsure
- Afraid
- Turned on/excited/ “freaky”/aroused (Note: use local term that connotes arousal)
- I refuse to answer

***She texts her best friend about the evening. What do you think she writes to her friend?***

1. ***________________________________________________________________________***

***________________________________________________________________________***

1. **How do you think P’s *mother* would react if she found out that her daughter spent the evening with a boy?**

- She would be angry
- She would be happy
- She would be worried
- She would not care
- I refuse to answer

**13. What would P’s *mother* do?**

- She would not allow P to see A
- She would threaten to punish P
- She would encourage P to see A again
- She would not do anything
- I refuse to answer

**14. How do you think P’s *father* would react if he found out that his daughter spent the evening with a boy?**

- He would be angry
- He would be happy
- He would be worried
- He would not care
- I refuse to answer

**15. What would P’s *father* do?**

- He would not allow P to see A again
- He would threaten or punish P
- He would encourage P to see A again
- He would not do anything
- I refuse to answer

VIGNETTE #2: CAN I GO OUT?

***One day after school, P and a few of her classmates, of whom some are boys and some are girls, decide to see a movie. The film will not end until 9 o’clock at night. Since she will get home late, she asks her mother’s permission to go to the movie. (Note: This is not about going to the movies but rather about going out in a mixed group. Modify both activity and return time as appropriate at your site.)***

**1. What do you think P will tell her mother in order to get her permission to go to the movie?**

- She tells her mother that she is *going with female and male friends*
- She tells her mother that she is *going only with female friends*
- She tells her mother she is going to the movies without telling her who she is going with
- She tells her mother that she is going to her classmate’s home to study
- She goes to the movie without telling her mother anything at all
- I refuse to answer

**2. What would you tell your mother in order to get her permission to go out with friends?**

- You would tell her that you are *going with female and male friends*
- You would tell her that you are *going only with female friends.*
- You would tell her you are going to the movies without telling her who you are going with
- You would tell her that you are going to a classmate’s home to study for the evening
- You would go to the movie without telling your mother anything at all
- I refuse to answer

***P tells her mother that she wants to see a movie and that she is going with her female and male friends.***

**3. What do you think her mother will do?**

- Refuse to let P go without saying why
- Refuse to let P go, and explain why she cannot go
- Refuses to let P go, but promises to take her to see the film another day
- Agree to let P go to the movie, on the condition that she picks P up when the movie is over
- Agree to let P go to the movie, on the condition that she or her older brother goes with her
- Agree to let P go to the movie, without any condition
- Tell her to speak with her father. She will agree if he does.
- I refuse to answer

***P’s mother refuses to let her go to the movie. P tries again and explains to her mother exactly with whom she is going, what film they will see, and guarantees to be back at a certain time. Her mother still does not agree to let her go.***

**4. Why do you think P’s mother refuses to let her go to the movie? Select the most important reason.**

- She is worried about P’s safety coming home after the movie by herself
- She is worried that P’s friends may be a bad influence
- She is worried that P will start going out with boys
- She is worried that the movie may not be suitable for young girls
- She is worried that going out will affect her schoolwork and grades
- She is worried about P’s father’s reaction
- I refuse to answer

**5. How do you think P feels when her mother refuses to let her go to the movie?**

- She feels embarrassed to tell her friends
- She feels angry at her mother
- She understands and respects her mother’s decision
- She feels sad/upset that her mother continues to treat her like a child
- She worries about what she is going to tell her friends
- I refuse to answer

**6. What do you think P will do when her mother refuses to let her go to the movie?**

- Obey her mother and not go to the movie
- Continue to beg her, hoping that she will change her mind
- Argue with her mother, saying that she does not understand what it is like to be a teenager today
- Try to get her father’s permission
- Ignore her mother and go to the movie anyway
- I refuse to answer

**7. What would you do in P’s situation?**

- Obey your mother
- Continue to beg your mother, hoping that she will change her mind
- Argue with your mother, saying that she does not understand what it is like to be a teenager today
- Try to get your father’s permission
- Ignore your mother and go to the movie anyway
- I refuse to answer

VIGNETTE #3: TEASING/ BULLYING

***Ever since she was a child, P has always felt bored playing with other girls, and prefers to play with boys. She is now 13 and one day after school, her male classmates are standing in a circle chatting in the middle of the playground. P wants to join them. She approaches them asking to join in.***

**1. What do you think her male classmates do when P asks to join in?**

- They welcome her to join, just like anyone else
- They say that she can’t join since the conversation is for boys only
- They refuse and call her names for wanting to talk about guy stuff
- They allow her to join because she is harmless
- They allow her to join because she is good at sports they play
- They allow her to join, but tease her
- I refuse to answer

***The boys refuse to allow P to join their conversation.***

**2. Why do you think they refuse to let her join their circle?**

- Because they think that girls should not want to talk about guy stuff
- Because they think girls should hang out with girls and boys with boys
- Because they think that P is very weird/unusual
- Because they don’t want to be friends with someone they think is a lesbian/homosexual
- I refuse to answer

***Conversation turns to sports and the boys start organizing a football game. Again, P asks to join. Again she is told no.***

**3. What do most of P’s female classmates think about her not being allowed to play football?**

- They think she should be able to play
- They think it is unfair, but girls are never allowed to play with boys
- They don’t understand why she even asked
- They think she is weird and just makes trouble for herself
- They think she is probably a lesbian/homosexual
- I refuse to answer

***Since P is not allowed to join the boys, she tries to join the girls’ group instead.***

**4. What do you think the other girls will do?**

- They welcome her just like anyone else
- They let her join the group, but ignore her
- They say that she can’t join because he is not a real girl
- They allow her to join them but tease her
- I refuse to answer

**5. What are most of P’s female classmates most likely to think of her for wanting to join boys’ conversations and games?**

- They admire her for acting as she wishes
- They think that P is weird
- They disapprove of P’s behavior
- They think that P is probably a lesbian/homosexual
- I refuse to answer

**6. All the girls and boys refuse to play with P. How do you think she feels?**

- She does not really care
- She is confused and does not understand why she cannot play
- She is angry that nobody likes her for who she is
- She is sad and feels rejected
- She is scared that something is wrong about her
- I refuse to answer

**7. How do you think P’s *mother* reacts to the fact that P behaves more like a boy than a girl?**

- Her mother tells P that she needs to start behaving “like a girl”
- Her mother encourages P to be who she wants to be
- Her mother is embarrassed by P
- Her mother worries what the neighbors will think
- Her mother doesn’t care about P’s behavior
- Her mother takes her to a doctor/local healer/pastor
- I refuse to answer

**8. How do you think P’s *father* reacts to her preference for boy things?**

- Her father says that P needs start acting “like a girl”
- Her father encourages P to be who she wants to be
- Her father is embarrassed by P
- Her father worries what the neighbors will think
- Her father takes her to a doctor/local healer/pastor
- Her father doesn’t care one way or another about P’s behavior
- I refuse to answer

VIGNETTE #4: PUBERTY

***P is 11 years old. Long before her friends she began to grow tall and then started to develop breasts. While she started to look like a woman all her friends continued to look like children. Just the other day she got her first menstrual period.***

**1. How do you think P feels about what’s going on with her body?**

- She is happy that she is becoming a grownup
- She is worried that her friends will tease her
- She thinks that she is sick and something is terribly wrong
- She is embarrassed about the changes she is experiencing
- She is really sad about starting periods
- She is confused and wants more information about the changes she is experiencing
- I refuse to answer

***P is confused about the changes that she is experiencing.***

1. **What does she do next?**

- Keep it a secret that she has started having her periods
- Speak with someone and ask for advice
- Search for information without talking to anyone
- Try to hide her body changes
- I refuse to answer

1. **She decides to speak with someone. Who would she speak with?**

- Her father
- Her mother
- Her grandmother
- A female friend
- A male friend
- Her older sister
- Her teacher
- Her aunt
- Her cousin
- I refuse to answer

***P tells her mother about her body changes***

1. **How does her mother *first* react to this news?**

- Her mother tells P she is happy for her
- Her mother says she is worried about what the changes mean for her
- Her mother teases P
- Her mother punishes her
- Her mother tells P that now that she is growing up, it is time to take on more responsibilities at home
- Her mother tells her that she now she should no longer play with boys
- Her mother tells her that she needs to be careful about how she dresses when she goes out of the house
- Her mother will teach her about hygiene and the meaning of periods
- I refuse to answer

1. **P knows that her mother has told her father that she has started periods. How does her father react to this news?**

- Her father tells P he is happy for her
- He says he is worried about what the changes mean for her
- Her father teases her
- Her father punishes her
- Her father tells P that now that she is growing up, it is time to take on more responsibilities at home
- Her father tells her that she now needs to be careful around boys
- Her father tells her that she needs to be careful about how she dresses when she goes out of the house
- Her father tells the mother to talk to P
- I refuse to answer

***On the way home from school P is walking with her friends and they notice that her breasts are showing under her dress.***

1. **What do you think they are most likely to do?**

- They will be jealous of her
- They will laugh and tease her
- They will offer to help cover her up
- They will be too embarrassed to say anything
- They will tell her that she better stay away from boys
- They will tell her it is time to get a boyfriend
- I refuse to answer

***P’s friends tease her and continue to tease her over the next few weeks. She is feeling more and more alone. She decides to speak with her older sister.***

1. **What is her sister most likely to say to her?**

- “You need to find other friends”
- “It is time that you found a boyfriend”
- “You need to stay at home more”
- “You should be proud that you are growing up”
- I refuse to answer

VIGNETTE #5: DRESSING APPROPRIATELY

***P has a favorite dress that she got one year ago. Lately her body has started to change – she is taller and has become more “curvy” as her breasts and hips are growing. The dress is now very short and tight on her. One day P’s mother sees P leaving the house in the dress and says that now that P is “becoming a woman, she shouldn’t be seen in public ‘looking like that.’”***

**1. What do you think P will do?**

- Ask her mother to explain what she means
- Change her clothes with no plans to wear the dress again
- Change her clothes but wear the dress when her mother is not around
- Argue with her mother
- Ignore her mother
- Tell her mother to buy her other clothes
- I refuse to answer

***P doesn’t understand… This is her favorite dress. P argues with her mother and asks why her dress is inappropriate.***

**2. What do you think P’s mother says to her?**

- “The dress is too short and tight on you”
- “Boys will get the wrong idea about you”
- “The dress show parts of your body that shouldn’t be shown”
- “Real women do not wear something like that”
- “Just do as I say and stop asking so many questions”
- I refuse to answer

***P hears what her mother is saying, but she does not think that there is a problem with her dress and the way she looks.***

**3. What would it take for her to give up her favorite dress?**

- If she can get a new dress
- If her female friends tease her
- If a boy she likes says something
- If her father is angry
- If she is called names at school or on the street
- I refuse to answer

***P does not argue with her mother. She changes her dress, but takes it with her and when she is out of the house she changes back. Her mother finds out.***

1. **What is P’s mother most likely to say when she finds out that P still wore the dress?**

- “This is an embarrassment”
- “Do you really want girls to get the wrong impression of you?”
- “Do you really want boys to be looking at you?”
- Nothing; her mother doesn’t really care
- I refuse to answer

1. **What do you think P’s mother is most likely to do?**

- She explains again and gives P one more chance
- She takes the dress away from P
- She punishes P by slapping, spanking or beating her
- She tells P’s father about the situation
- I refuse to answer

VIGNETTE #6 #Optional#: PREGNANCY

***P is 14 years old and in 9^th^ grade (or appropriate grade per site). Her boyfriend, A, is also 14 years old. Lately P has been feeling sick. She has missed periods and now realizes that she is pregnant, and that A is responsible for the pregnancy. P can’t believe it. Her best friend notices that P is not herself and asks her what the problem is.***

1. **How do you think P is *feeling*?**

- Confused
- Scared
- Happy
- Angry
- Very sad
- I refuse to answer

1. **How do you think P is most likely to react when she realizes that she is pregnant?**

- Be happy that she is having a baby with A
- Deny that she is pregnant
- Deny that A is responsible for the pregnancy
- Accuse A of trapping her into being pregnant
- Accept that she is pregnant but refuse any further involvement with A
- I refuse to answer

1. **How do you think you would react if you were ever in P’s situation?**

- I would be happy that I was having a baby with A
- Deny that I am pregnant
- Deny that A is responsible for the pregnancy
- I would accuse A of trapping me into being a mother
- I would accept that I am pregnant but refuse any further involvement with A
- I refuse to answer

***P tells her friend that she is pregnant and that A has caused the pregnancy.***

**4. What is her friend most likely to advise P to do?**

- Run away from home
- Ask her boyfriend to take responsibility and marry her as soon as they can
- Give the child up for adoption
- “Be a woman” and take responsibility
- Have an abortion
- I refuse to answer

***P is scared and decides to run away from home. She tells her friend of her plan, who in turn tells P’s younger sister. Her younger sister then tells their parents that P is pregnant and A is responsible.***

1. **How will P’s parents react when they find out that their daughter is pregnant? They will…**

- Accuse A of tricking P into having sex
- Kick P out of the house
- Say that they will find the money for P to have an abortion
- Say they will force P to marry A as soon as possible
- Say they will take care of the baby no matter what P decides to do with A
- I refuse to answer

***P’s parents insist that their daughter is not responsible for the pregnancy. They say that P is too young and “innocent” to be having sex, and accuse A of tricking their daughter into having sex with him.***

1. **What will P do next?**

- She denies responsibility
- She decides to have the baby but breaks the relationship with A
- She decides she will have the baby and marry A as soon as she can
- She gets an abortion
- She runs away from home
- She does nothing and just waits to see what happens next
- I refuse to answer

***P thinks she should end the pregnancy and finds a place where she can have an abortion at a good price.***

1. **What is P most likely to do next?**

- Change her mind at the last minute and continue the pregnancy/keep the baby
- End her relationship with A and continue the pregnancy alone
- Demand money for an abortion from A, but deal with it alone
- Demand that A accompany her to have the abortion
- I refuse to answer

1. **What do *you* think P *should* do in this situation?**

- Continue the pregnancy/keep the baby
- End her relationship with A and continue the pregnancy alone
- Demand money for an abortion from A, but deal with it alone
- Demand that A accompany her to have the abortion
- I refuse to answer

1. **What do you think *your friends would do* if they ever were in this kind of situation?**

- Continue the pregnancy/keep the baby
- End the relationship with A and continue the pregnancy alone
- Demand money for an abortion from A, but deal with it alone
- Demand that A accompany her to have the abortion
- I refuse to answer
