# Supplementary material for: Measuring young adolescent perceptions of relationships: A vignette-based approach to exploring gender equality
Source: PLoS One. 2019 Jun 27;14(6):e0218863. doi: 10.1371/journal.pone.0218863 (PMC6597075; doi:10.1371/journal.pone.0218863)
Supplement: S4 Text — (DOCX) [file pone.0218863.s006.docx]

**S4 Text.**

**Vignettes Instrument (Repilot)**

**The Global Early Adolescent Study**

VIGNETTE #1: PROTAGONIST (P) LIKES ANTAGONIST (A)

VIGNETTE #2: TEASING/ BULLYING

VIGNETTE #3: PUBERTY

VIGNETTE #4: GIRLS WHO PREFER TO PLAY WITH BOYS

VIGNETTE #5: PREGNANCY

**MALE VERSION**

VIGNETTE #1: PROTAGONIST (P) LIKES ANTAGONIST (A)

***P is in your grade. He is attracted to A, who is in the same grade, but he doesn’t know her and has never spoken with her in person. Most of his friends say they have girlfriends but he has never had one before. He wants to get her attention, but is not sure how.***

1. **What do you think he is most likely to do?**

- Ask a friend to tell A that P likes her
- Pass her a note
- Go up and talk to her directly
- Nothing, just wait hoping he will meet her
- I refuse to answer

1. **What about you? What do you think you would do in that situation?**

- Ask a friend to tell A that you like her
- Pass A a note
- Go up and talk to A directly
- Nothing, just wait hoping you will meet her
- I refuse to answer

***P decides to talk to A himself. He is walking out of school with his friends when they run into a group of girls that includes A. They all start talking about a friend’s party that is happening next Friday. P wants to know if A is going, but is afraid to ask. [NOTE: can be modified to be site-specific event. What is important is that they have an event where boys and girls will get together.]***

1. **What do you think he is most likely to do?**

- Ask if any of the girls are going to the party
- Ask A directly if she is going to the party
- Get a friend to ask A if she is going to the party
- Say nothing and hope that someone else will ask A if she is going
- Ask A’s friend if she knows if A is going to the party
- I refuse to answer

1. **What do you think you would do if you were in that situation?**

- Ask if any of the girls are going to the party
- Ask A directly if she is going to the party
- Get a friend to ask A if she is going to the party
- Say nothing and hope that someone else will ask A if she is going
- Ask A’s friend if she knows if A is going to the party
- I refuse to answer

***It is Friday, and P and A are both at the party with their friends. P sees that A is standing in a corner across the room. What would it take for him to talk to her?***

1. **He would talk to her if:**

- His friends challenged or encouraged him to go up to A
- A was alone
- A came up to him or noticed him in another way
- He knew from a friend that A liked him
- He would not talk to A under any circumstance
- I refuse to answer

1. **Version A. As it turns out one of A’s friends tells P that she likes him. Knowing this, what do you think he will do?**

- Go up and speak with A directly
- Do nothing and hope A will notice him
- Give “a look”
- Walk by and bump into A “accidentally”
- Ignore A
- I refuse to answer

**Version B. As it turns out P’s friends tell him that it is inappropriate for someone his age to be interested in girls. What do you think P will do?**

- Go up and speak with A directly
- Do nothing and hope A will notice him
- Give “a look”
- Walk by and bump into A “accidentally”
- Ignore A
- I refuse to answer

1. **In P’s situation and you knew that A liked you, what do you think you would do?**

- Go up and speak with A directly
- Do nothing and hope A will notice him
- Give “a look”
- Walk by and bump into A “accidentally”
- Ignore A
- I refuse to answer

***It is also quite possible that P might not talk with A at all.***

1. **What is the most likely reason that he might *not* talk to her?**

- He is too embarrassed/shy
- He is afraid of her rejection
- He would be teased by his friends if he did so
- He has been told by parents that it is not appropriate to talk with a girl directly
- There is no reason why P would not talk to A
- I refuse to answer

1. **If you were in the same situation, why might you not talk to her?**

- I would be too embarrassed/shy
- I would be afraid of rejection by the girl
- I would be afraid I would be teased by my friends
- I would be afraid because I am not allowed to have a girlfriend
- I can’t think of a reason why I would not talk to her
- I refuse to answer

***P and A did talk. They exchanged phone numbers, and P is now back home and thinks back on his evening.***

1. **How is he feeling? (pick the one that best describes his feeling)**

- Happy
- Proud
- Nervous
- Unsure
- Afraid
- I refuse to answer

VIGNETTE #2: TEASING/ BULLYING

***Ever since he was a child, P has always felt bored playing with other boys, and prefers to play with girls. He is now 13 and one day after school he sees his female classmates standing in a circle chatting in the middle of the playground. P wants to join them. He approaches them asking to join in.***

1. **What do you think his female classmates do when P asks to join in?**

- They welcome him to join, just like anyone else
- They say that he can’t join since the conversation is for girls only
- They refuse and call him names for wanting to talk about girl stuff
- They allow him to join because he is harmless
- They allow him to join because he is good at the games they play
- They allow him to join, but tease him
- I refuse to answer

***The girls refuse to allow P to join their conversation.***

1. **Why do you think they refuse to let him join their circle?**

- Because they feel uncomfortable discussing girl stuff with a boy
- Because they think boys should hang out with boys and girls with girls
- Because they think that P is very weird/unusual
- Because they don’t want to be friends with someone they think is gay/homosexual
- I refuse to answer

***Conversation turns to jump rope (popular local game that girls play). Again, P asks to join. Again he is told no.***

**What do most of P’s male classmates think about him not being allowed to make join the girls’ game?**

They think he should be able to participate

They think it is unfair, but boys are never allowed to play with girl

They think he is weird and just makes trouble for himself

They think he is probably gay/homosexual

I refuse to answer

***Since P is not allowed to join the girls, he tries to join the boys’ group instead.***

1. **What do you think the other boys will do?**

- They welcome him, just like anyone else
- They let him join the group, but ignore him
- They say that he can’t join because he is not a real boy
- They allow him to join them but tease him
- I refuse to answer

1. **What are most of P’s male classmates most likely to think of him for wanting to join girls’ conversations and activities?**

- They admire him for acting as he wishes
- They think that P is weird
- They disapprove of P’s behavior
- They think that P is probably gay/homosexual
- I refuse to answer

1. **What do you think you would do if you were with your classmates and P wanted to join?**

- Welcome him, just like anyone else
- Let him join the group, but ignore him
- Say that he can’t join because he is not a real boy
- Allow him to join but make fun of him
- I refuse to answer

1. **All the boys and girls refuse to play with P. How do you think he feels?**

- He does not really care
- He is confused and does not understand why he cannot play
- He is angry that nobody likes him for who he is
- He is sad and feels rejected
- He is scared that something is wrong about him
- I refuse to answer

1. **How do you think P’s *mother* reacts to the fact that P prefers to play with girls?**

- His mother tells P that he needs to start behaving “like a boy”
- His mother encourages P to be who he wants to be
- His mother is embarrassed by P
- His mother worries what the neighbors will think
- His mother takes him to a doctor/local healer/pastor
- His mother doesn’t care one way or another about P’s behaviour
- I refuse to answer

1. **How do you think P’s *father* reacts to the fact that P prefers to play with girls?**

- His father says that P needs start acting “like a boy”
- His father encourages P to be who he wants to be
- His father is embarrassed by P
- His father worries what the neighbors will think
- His father takes him to a doctor/local healer/pastor
- His father doesn’t care one way or another about P’s behaviour
- I refuse to answer

VIGNETTE #3: PUBERTY

***P is 15 years old. He has been worried for a long time that all the other boys in his group were growing taller and that they also had some facial hair. Until recently, P had seen none of those changes himself. The other morning he woke up and his underwear was stained because he had a ‘wet dream.’***

1. **What is P most likely to feel about the body changes that he is experiencing, and the fact that he is going through puberty?**

- He is happy that he is becoming a grownup
- He thinks that he is sick and something is terribly wrong
- He is embarrassed about the changes he is experiencing
- He is worried about the changes
- He is sad about becoming an adult
- He is confused and wants more information about the changes he is experiencing
- I refuse to answer

***P is confused about the changes that he is experiencing.***

1. **What is he most likely to do next?**

- Tell no one that he has finally started puberty
- Speak with someone about his concerns
- Search for information without talking to anyone
- Try to hide his body changes
- I refuse to answer

***P tells his father about his body changes.***

1. **How is his father most likely to *first* react that he has finally begun puberty?**

- His father tells P he is happy now that he is becoming a man
- His father makes fun of him
- His father tells him that now that he is growing up, it is time to take on more responsibilities at home
- His father tells him that now he should no longer play with girls
- His father will teach him about hygiene and about the meaning of wet dreams/nocturnal emission
- I refuse to answer

***For years, P has been the subject of jokes and teasing by his more mature guy friends. Now they start seeing that P is also maturing.***

1. **What do you think they are most likely to do?**

- They will make fun of him for being slow
- They will be too embarrassed to say anything
- They will tell him now that he is a man it is time to get a girlfriend
- They will see it as normal and pay no attention to it
- I refuse to answer

***P’s friends tease him for being slower than everyone else to enter puberty, and continue to tease him over the next few weeks. He is feeling more and more alone. He decides to speak with his older brother.***

1. **What is his brother most likely to say to him?**

- “Ignore them”
- “You are a man now go find a girlfriend”
- “You need to stay at home more”
- “You should be proud that you are growing up”
- His brother probably won’t say anything
- I refuse to answer

VIGNETTE #4: GIRLS WHO PREFER TO PLAY WITH BOYS

***Ever since she was a child, P has always felt bored playing with other girls, and prefers to play with boys. She is now 13 and one day after school she sees her male classmates standing in a circle chatting in the middle of the playground. P wants to join them. She approaches them asking to join in.***

1. **What do you think the boys would do when P asks to join in?**

- They welcome her to join, just like anyone else
- They say that she can’t join since the conversation is for boys only
- They refuse and tease her for wanting to act like a boy
- They allow her to join because she isn’t girlish
- They allow her to join, but tease her because she is a girl
- They allow her to join, but tease her because she acts like a boy
- I refuse to answer

***The boys refuse to allow P to join their conversation.***

1. **If you were with these boys, would you agree with their decision?**

- Yes, because she would of caused trouble in the group
- Yes, because she is weird and you prefer keeping her at a distance
- Yes, because she would have been bullied if she had joined
- No, you would have let her join like anyone else
- No, you would have let her join but would have kept her at a distance
- I refuse to answer

1. **Why do you think the boys would refuse to let her join their circle?**

- Because they feel uncomfortable discussing boy stuff with a girl
- Because they think boys should hang out with boys and girls with girls
- Because they think that P is very weird/unusual
- Because they don’t want to be friends with someone they think is gay/homosexual
- I refuse to answer

VIGNETTE#5: PREGNANCY

***P is 14 years old and in 9^th^ grade (or appropriate grade per site). His girlfriend, A, is also 14 years old. Recently, A realized that she is pregnant, and told P that made her pregnant. The next day, P’s best friend notices that he is not himself and asks him what the problem is.***

1. **How do you think P is *feeling*? *(pick the one best choice)***

- Scared
- Happy
- Proud
- Angry
- Sad
- I refuse to answer

1. **How do you think P is most likely to react to A’s pregnancy?**

- Angry with A that she got pregnant
- Deny that he had anything to do with A getting pregnant
- Accuse A of trapping him into being a father
- Accepts the pregnancy but refuses any further involvement with A
- Depends on how he feels about A
- I refuse to answer

1. **How do you think you would react if you were ever in P’s situation?**

- You would hope that the problem would go away on its own and would do nothing
- You would be happy that you were having a baby with A
- You would deny that you were responsible for the pregnancy
- You would accuse A of trapping you into being a father
- You would accept that A is pregnant but refuse any further involvement with her
- I refuse to answer

***P tells his friend that his girlfriend A is pregnant and that he has caused the pregnancy.***

1. **What is his friend most likely to advise P to do?**

- Run away from home
- Tell A to have the baby and raise it
- Tell A have the baby but give it up for adoption
- “Be a man” and take responsibility
- Tell A to get an abortion
- Assume equal responsibility with A and make a joint decision
- Do whatever A decides
- I refuse to answer

***P is scared and plans to run away from home. He tells his younger sister. His younger sister tells their parents that P’s girlfriend is pregnant and P is responsible.***

1. **How will P’s parents react when they find out that their son’s girlfriend is pregnant? They will…**

- Accuse A of tricking P into having sex
- Kick P out of the house
- Say that they will find the money for A to have an abortion
- Say they will force P to marry A as soon as possible
- Say they will take care of the baby no matter what P decides to do with A
- I refuse to answer

***P’s parents insist that their son is not responsible for the pregnancy. They say that P is too young and “innocent” to be having sex, and accuse A of tricking their son into having sex with her.***

1. **What will P do next?**

- He denies responsibility
- He decides to end the relationship with A after the baby is born
- He marries A as soon as he can
- He helps A get an abortion
- He runs away from home
- He does nothing and just waits to see what happens next
- I refuse to answer

***P thinks A should end the pregnancy and finds a place where A can have an abortion at a good price.***

1. **What is P most likely to do next?**

- Ask A to continue the pregnancy/keep the baby
- End his relationship with A and let her continue the pregnancy alone
- Help A get money for an abortion, but leave her to deal with it alone
- Accompany A to have the abortion
- Try to force A to have an abortion, but provide no help
- I refuse to answer

1. **What do *you* think P *should* do in this situation? What would be the right thing to do?**

- Ask A to continue the pregnancy/keep the baby
- End his relationship with A and let her continue the pregnancy alone
- Help A get money for an abortion, but leave her to deal with it alone
- Accompany A to have the abortion
- Try to force A to have an abortion, but provide no help
- I refuse to answer

1. **What do you think *your friends would do* if they ever were in this kind of situation?**

- Ask A to continue the pregnancy/keep the baby
- End his relationship with A and let her continue the pregnancy alone
- Help A get money for an abortion, but leave her to deal with it alone
- Accompany A to have the abortion
- Try to force A to have an abortion, but provide no help
- I refuse to answer

**FEMALE VERSION**

VIGNETTE #1: PROTAGONIST (P) LIKES ANTAGONIST (A)

VIGNETTE #2: TEASING/ BULLYING

VIGNETTE #3: PUBERTY

VIGNETTE #4: BOYS WHO PREFER TO PLAY WITH GIRLS

VIGNETTE #5: PREGNANCY

VIGNETTE #1: PROTAGONIST (P) LIKES ANTAGONIST (A)

***P is in your grade. She is attracted to A, who is in the same grade, but she doesn’t know him and has never spoken with him in person. Most of her friends say they have boyfriends but she has never had one before. She wants to get his attention, but is not sure how.***

1. **What do you think she is most likely to do?**

- Ask a friend to tell A that P likes him
- Pass him a note
- Go up and talk to him directly
- Nothing, just wait hoping she will meet him
- I refuse to answer

1. **What about you? What do you think you would do in that situation?**

- Ask a friend to tell A that you like him
- Pass A a note
- Go up and talk to A directly
- Nothing, just wait hoping you will meet him
- I refuse to answer

***P decides to talk to A herself. She is walking out of school with her friends when they run into a group of boys that includes A. They all start talking about a friend’s party that is happening next Friday. P wants to know if A is going, but is afraid to ask. (NOTE: can be modified to be site-specific event. What is important is that they have an event where boys and girls will get together.)***

1. **What do you think she is most likely to do?**

- Ask if any of the boys are going to the party
- Ask A directly if he is going to the party
- Get a friend to ask A if he is going to the party
- Say nothing and hope that someone else will ask A if he is going
- Ask A’s friend if he knows if A is going to the party
- I refuse to answer

1. **What do you think you would do if you were in that situation?**

- Ask if any of the boys are going to the party
- Ask A directly if he is going to the party
- Get a friend to ask A if he is going to the party
- Say nothing and hope that someone else will ask A if he is going
- Ask A’s friend if he knows if A is going to the party
- I refuse to answer

***It is Friday, and P and A are both at the party with their friends. P sees that A is standing in a corner across the room. What would it take for her to talk to him?***

1. **She would talk to him if:**

- Her friends challenged or encouraged her to go up to A
- A was alone
- A came up to her or noticed her in another way
- She knew from a friend that A liked her
- She would not talk to A under any circumstance
- I refuse to answer

1. **Version A. As it turns out one of A’s friends tells P that he likes her. Knowing this, what do you think she will do?**

- Go up and speak with A directly
- Do nothing and hope A will notice her
- Give “a look”
- Walk by and bump into A “accidentally”
- Ignore A
- I refuse to answer

**Version B: As it turns out P’s friends tell her that it is inappropriate for someone her age to be interested in boys. What do you think P will do?**

- Go up and speak with A directly
- Do nothing and hope A will notice her
- Give “a look”
- Walk by and bump into A “accidentally”
- Ignore A
- I refuse to answer

1. **If you were in P’s situation and you knew that A liked you, what do you think you would do?**

- Go up and speak with A directly
- Do nothing and hope A will notice me
- Give “a look”
- Walk by and bump into A “accidentally”
- Ignore A
- I refuse to answer

***It is also quite possible that P might not talk with A at all.***

1. **What is the most likely reason that she might *not* talk to him?**

- She is too embarrassed/shy
- She is afraid of his rejection
- She would be teased by her friends if she did so
- She worries about being scolded because she has been told to stay away from boys
- There is no reason why P would not talk to A
- I refuse to answer

1. **If you were in the same situation, why might you not talk to him?**

- I would be too embarrassed/shy
- I would be afraid of rejection by the boy
- I would be afraid I would be teased by my friends
- I would be afraid because I am not allowed to have a boyfriend
- I can’t think of a reason why I would not talk to him
- I refuse to answer

***P and A did talk. They exchanged phone numbers, and P is now back home and thinks back on her evening.***

1. **How is she feeling? (Pick the one that best describes her feeling.)**

- Happy
- Proud
- Nervous
- Unsure
- Afraid
- I refuse to answer

VIGNETTE #2: TEASING/ BULLYING

***Ever since she was a child, P has always felt bored playing with other girls, and prefers to play with boys. She is now 13 and one day after school she sees her male classmates standing in a circle chatting in the middle of the playground. P wants to join them. She approaches them asking to join in.***

1. **What do you think her male classmates do when P asks to join in?**

- They welcome her to join, just like anyone else
- They say that she can’t join since the conversation is for boys only
- They refuse and call her names for wanting to talk about guy stuff
- They allow her to join because she is harmless
- They allow her to join because she is good at the games they play
- They allow her to join, but tease her
- I refuse to answer

***The boys refuse to allow P to join their conversation.***

1. **Why do you think they refuse to let her join their circle?**

- Because they feel uncomfortable discussing boy stuff with a girl
- Because they think girls should hang out with girls and boys with boys
- Because they think that P is very weird/unusual
- Because they don’t want to be friends with someone they think is gay/homosexual
- I refuse to answer

***Conversation turns to football [site-specific: popular local game that boys play]. Again, P asks to join. Again she is told no.***

**What do most of P’s female classmates think about her not being allowed to join the boys’ game?**

They think she should be able to participate

They think it is unfair, but girls are never allowed to play with boys

They think she is weird and just makes trouble for herself

They think she is probably gay/homosexual

I refuse to answer

***Since P is not allowed to join the boys, she tries to join the girls’ group instead.***

1. **What do you think the other girls will do?**

- They welcome her, just like anyone else
- They let her join the group, but ignore her
- They say that she can’t join because she is not a real girl
- They allow her to join them, but tease her
- I refuse to answer

1. **What are most of P’s female classmates most likely to think of her for wanting to join boys’ conversations and activities?**

- They admire her for acting as she wishes
- They think that P is weird
- They disapprove of P’s behaviour
- They think that P is probably gay/homosexual
- I refuse to answer

1. **What do you think you would do if you were with your classmates and P wanted to join?**

- Welcome her, just like anyone else
- Let her join the group, but ignore her
- Say that she can’t join because she is not a real girl
- Allow her to join, but make fun of her
- I refuse to answer

1. **All the girls and boys refuse to play with P. How do you think she feels?**

- She does not really care
- She is confused and does not understand why she cannot play
- She is angry that nobody likes her for who she is
- She is sad and feels rejected
- She is scared that something is wrong about her
- I refuse to answer

1. **How do you think P’s *mother* reacts to the fact that P prefers to play with boys?**

- Her mother tells P that she needs to start behaving “like a girl”
- Her mother encourages P to be who she wants to be
- Her mother is embarrassed by P
- Her mother worries what the neighbours will think
- Her mother takes her to a doctor/local healer/pastor
- Her mother doesn’t care one way or another about P’s behaviour
- I refuse to answer

1. **How do you think P’s *father* reacts to the fact that P prefers to play with boys?**

- Her father says that P needs start acting “like a girl”
- Her father encourages P to be who she wants to be
- Her father is embarrassed by P
- Her father worries what the neighbours will think
- Her father takes her to a doctor/local healer/pastor
- Her father doesn’t care one way or another about P’s behaviour
- I refuse to answer

VIGNETTE #3: PUBERTY

***P is 15 years old. She has been worried for a long time that all the other girls in her group were becoming curvier and starting to develop breasts. Until recently, P had seen none of those changes herself. The other day she got her first period; and she has started noticing hair where she didn’t have it before.***

1. **What is P most likely to feel about the body changes she is experiencing, and the fact that she is going through puberty?**

- She is happy that she is becoming a grownup
- She thinks that she is sick and something is terribly wrong
- She is embarrassed about the changes she is experiencing
- She is worried about the changes
- She is sad about becoming an adult
- She is confused and wants more information about the changes she is experiencing
- I refuse to answer

***P is confused about the changes that she is experiencing.***

1. **What is she most likely to do next?**

- Tell no one that she has finally started puberty
- Speak with someone about her concerns
- Search for information without talking to anyone
- Try to hide her body changes
- I refuse to answer

***P tells her mother about her body changes.***

1. **How is her mother most likely to *first* react to the fact that P has finally begun puberty?**

- Her mother tells P she is happy now that she is becoming a woman
- Her mother makes fun of her
- Her mother tells her that now that she is growing up, it is time to take on more responsibilities at home
- Her mother tells her she should no longer play with boys
- Her mother will teach her about hygiene and about the meaning of periods
- I refuse to answer

***For years, P has been the subject of jokes and teasing by her more mature girl friends. Now they start seeing that P is also maturing.***

1. **What do you think they are most likely to do?**

- They will make fun of her for being slow
- They will be too embarrassed to say anything
- They will tell that because her now that she is a woman, it is time to get a boyfriend
- They will see it as normal and pay no attention to it
- I refuse to answer

***P’s friends tease her for being slower than everyone else to enter puberty, and continue to tease her over the next few weeks. She is feeling more and more alone. She decides to speak with her older sister.***

1. **What is her sister most likely to say to her?**

- “Ignore them”
- “You are a woman now; go find a boyfriend”
- “You need to stay at home more”
- “You should be proud that you are growing up”
- Her sister probably won’t say anything
- I refuse to answer

VIGNETTE#4: BOYS WHO PREFER TO PLAY WITH GIRLS

***Ever since he was a child, P has always felt bored playing with other boys, and prefers to play with girls. He is now 13, and one day after school he sees his female classmates standing in a circle chatting in the middle of the playground. P wants to join them. He approaches them asking to join in.***

1. **What do you think the girls would do when P asks to join in?**

- They welcome him to join, just like anyone else
- They say that he can’t join since the conversation is for girls only
- They refuse and tease him for wanting to act like a girl
- They allow him to join because he isn’t boy-ish
- They allow him to join, but tease him because he is a boy
- They allow him to join, but tease him because he acts like a girl
- I refuse to answer

***The girls refuse to allow P to join their conversation.***

1. **If you were with these girls, would you agree with their decision?**

- Yes, because he would of caused trouble in the group
- Yes, because he is weird and you prefer keeping him at a distance
- Yes, because he would have been bullied if he had joined
- No, you would have let him join like anyone else
- No, you would have let him join, but would have kept him at a distance
- I refuse to answer

1. **Why do you think the girls would refuse to let him join their circle?**

- Because they feel uncomfortable discussing girl stuff with a boy
- Because they think girls should hang out with girls and boys with boys
- Because they think that P is very weird/unusual
- Because they don’t want to be friends with someone they think is gay/homosexual
- I refuse to answer

VIGNETTE#5: PREGNANCY

***P is 14 years old and in 9^th^ grade [or appropriate grade per site]. Her boyfriend, A, is also 14 years old. Recently, P realized that she is pregnant, and told A that he had made her pregnant. The next day, P’s best friend notices that she is not herself and asks her what the problem is.***

1. **How do you think P is *feeling*? *(Pick the one best choice)***

- Scared
- Happy
- Proud
- Angry
- Sad
- I refuse to answer

1. **How do you think P is most likely to react to realizing that she is pregnant?**

- Angry that A got her pregnant
- Deny that she is pregnant
- Accuse A of trapping her into being a mother
- Accept the pregnancy but refuses any further involvement with A
- Depends on how she feels about A
- I refuse to answer

1. **How do you think you would react if you were ever in P’s situation?**

- You would hope that the pregnancy would go away on its own and do nothing
- You would be happy to have a baby with A
- You would be happy to have a baby but would not want A involved
- You would not want to have a baby with A
- You would not want a baby with anyone at this time in your life
- I refuse to answer

***P tells her friend that she is pregnant and that A has caused the pregnancy.***

1. **What is her friend most likely to advise P to do?**

- Run away from home
- Have the baby and raise it
- Have the baby and give it up for adoption
- Assume equal responsibility with A and make a joint decision
- Get an abortion
- Do whatever A decides
- I refuse to answer

***P is scared and plans to run away from home. She tells her younger sister. Her younger sister tells their parents that P is pregnant and A is responsible.***

1. **How will P’s parents react when they find out that their daughter is pregnant? They will…**

- Accuse A of tricking P into having sex
- Kick P out of the house
- Say that they will find the money for A to have an abortion
- Say they will force P to marry A as soon as possible
- Say they will take care of the baby no matter what P decides to do with A
- I refuse to answer

***P’s parents insist that their daughter is not responsible for the pregnancy. They say that P is too young and “innocent” to be having sex, and accuse A of tricking their daughter into having sex with him.***

1. **What will P do next?**

- She denies responsibility
- She decides to end the relationship with A after the baby is born
- She marries A as soon as she can
- She gets an abortion
- She runs away from home
- She does nothing and just waits to see what happens next
- I refuse to answer

***P thinks she should end the pregnancy and finds a place where she can have an abortion at a good price.***

1. **What is P most likely to do next?**

- Continue the pregnancy/keep the baby
- End her relationship with A and continue the pregnancy alone
- Ask A for help getting money for an abortion, but deal with it alone
- As A to accompany her to have the abortion
- I refuse to answer

***If P decides she wants to continue the pregnancy…***

1. **What will she do next?**

- Tell her parents and go away to have the baby secretly
- Go away to have the baby secretly without telling her family
- Stay and have the baby with her family’s support
- Stay and have the baby with A’s support
- I refuse to answer

1. **What do *you* think P *should* do in this situation? What would be the right thing to do?**

- Continue the pregnancy/keep the baby
- End her relationship with A and continue the pregnancy alone
- Ask A for help getting money for an abortion, but deal with it alone
- Ask A to accompany her to have the abortion
- I refuse to answer

1. **What do you think *your friends would do* if they ever were in this kind of situation?**

- Continue the pregnancy/keep the baby
- End her relationship with A and continue the pregnancy alone
- Ask A for help getting money for an abortion, but deal with it alone
- Ask A to accompany her to have the abortion
- I refuse to answer
