# Supplementary material for: Measuring young adolescent perceptions of relationships: A vignette-based approach to exploring gender equality
Source: PLoS One. 2019 Jun 27;14(6):e0218863. doi: 10.1371/journal.pone.0218863 (PMC6597075; doi:10.1371/journal.pone.0218863)
Supplement: S5 Text — (DOCX) [file pone.0218863.s007.docx]

**S5 Text.**

**The GEAS Field Coordinator Survey**

**Vignettes Measures**

1. **Vignettes Workshop**
2. Did you hold a vignettes development workshop in your site?

❑ No.

❑ Yes. If yes:

1. Did you train those leading the vignettes workshop on the process?

❑ No.

❑ Can’t recall.

❑ Yes.

1. What setting did you use for the workshop?

______________________________________________________________________

1. How were the participants selected?

______________________________________________________________________

1. How many participants were there in the workshop? (write in the number): ______
2. How were the themes or “stems” of the vignettes decided upon? How did the workshop decide which themes or “stems” were most important?

1. What challenges, if any, did you face when conducting the vignette development workshop (this may include: selecting participants, identifying a venue for the workshop, getting parent permission, conflicts during the workshop itself or anything else that you may recall)? ❑ None.

Challenges faced:

1. Were there particular situations or stories that stick out in your mind from the workshop? ❑ None.

Situations/Stories:

1. What would you change were you to do the workshop over again? ❑ Nothing.

Changes I would make:

1. What lessons learned or best practices would you share with others doing this now or in the future? Please explain:

1. Overall, what do you think went well with the workshop and what problems did you face (such as specific scenarios, workshop participant relationships, role plays, etc.)?
2. What went well?
3. What was a problem?
4. Who wrote the draft vignette at your site? Can you tell us about youth participation in drafting notes and reviewing them?
5. **Development of core vignettes**
6. To what extent do you feel that your site had a voice in developing the overall core vignettes that were piloted as part of the GEAS Instruments?

❑ Not at all. ❑ Not much. ❑ A little. ❑ Some. ❑ A lot.

1. To what extent do you feel that the work your site did/your sites suggestions was reflected/incorporated in the core draft vignettes?

❑ Not at all. ❑ Not much. ❑ A little. ❑ Some. ❑ A lot.

1. What recommendations would you have for development of the core vignettes if the process were to be done over? ❑ None.

Recommendations:

1. **Vignettes piloting**
2. Were there issues your data collectors faced in piloting the vignettes or specific concerns young people faced in responding to the vignettes?

❑ No.

❑ Yes. If yes, what were they?

1. Who appeared to have the greatest difficulty understanding and/or responding to the vignettes (check all that apply)?

❑ No one.

❑ Younger (10-12) adolescents.

❑ Older Adolescents (13-14).

❑ No difference.

❑ Boys.

❑ Girls.

❑ No difference.

1. If participants experienced any difficulty, can you describe the issue? ________________________________________________________________________

________________________________________________________________________

1. If you observed any overarching differences that stand out to you in terms of how boys versus girls, and/or younger versus older adolescents responded to specific vignettes, please briefly describe what you observed:________________________________________________________________

________________________________________________________________________

1. Finally, are there any other observations that you have regarding the vignettes or the piloting?

Please explain: ___________________________________________________________

________________________________________________________________________
