# Supplementary material for: Measuring young adolescent perceptions of relationships: A vignette-based approach to exploring gender equality
Source: PLoS One. 2019 Jun 27;14(6):e0218863. doi: 10.1371/journal.pone.0218863 (PMC6597075; doi:10.1371/journal.pone.0218863)
Supplement: S6 Text — (DOCX) [file pone.0218863.s008.DOCX]

**S6 Text.**

**Additional Detail on the Field Coordinator Survey**

**Overview of The Field Coordinator Survey**

The field coordinator survey included three domains (vignettes workshop, development of core vignettes, vignette piloting) to assess each site’s experience with the development of the vignettes measures, and to identify challenges encountered during the process of administering the vignettes. A semi-structured questionnaire was designed to elicit thorough feedback, and distributed to the sites in October 2017.

Fourteen surveys, one per site, were distributed. Our team received feedback from nine sites (response rate: 64%). Eight out of nine sites submitted a complete survey and one site submitted a partially completed survey.

**Vignettes Workshop**

**Workshop Conducted and Training Provided:**

All nine sites indicated they had completed a vignettes development workshop, which included multiple sessions across three consecutive days. Eight out of nine sites reported proper training provided to personnel leading the workshop, and seven out of eight gave details about training programs offered. The types of training sites reported varied. Four out of seven sites reported focused training on study protocol/guidelines regarding conducting vignettes studies among adolescents; three indicated training on vignettes methods; four sites offered training on roleplays, which played an important role in collecting vignettes measure response options from young adolescents. Two sites also conducted simulated roleplays or interviews to ensure data collectors were equipped with the necessary facilitation skills.

**Participants Selection for Roleplay Session during Workshop:**

The participants for the workshops were selected purposively. Two sites received help from local NGOs to identify potential workshop candidates. In Nairobi eligible candidates were first identified through in-depth interviews, from which “talkative” and enthusiastic adolescents were invited to join the workshop. In other settings, participants were selected to represent diversity in family backgrounds, individual characteristics, religion, schools, and ethnic groups.

**Number of Adolescent Participants:**

An average of 12-15 adolescents were selected to join the vignettes workshop in each site, with an even divide between boys and girls. One site (Kinshasa) had 36 participants.

**Development of Vignettes Themes:**

Six sites reported that adolescents’ engagement played an essential role in vignette themes development, specifically in narrative interviews and roleplays. All feedback from adolescents directly influenced the ultimate content of the vignettes.

**Drafting of Vignettes:**

Field coordinators and research assistants from over half of the sites wrote the initial vignette drafts. Principal Investigators from the other sites (Ghent, Nairobi, and Baltimore) drafted the first version of vignettes measures. In Ouagadougou, interviewers took the lead developing the vignettes, incorporating adolescents’ feedback.

**Development of Core Vignettes**

**The Extent of Site Partners’ Engagement in Core Vignettes Development:**

An individual multiple-choice question with Likert Scale responses assessed partners’ engagement in the process (Question: “To what extent do you feel that your site had a voice in developing the overall core vignettes that were piloted as part of the GEAS Instruments?”; response options: not at all, not much, a little, some, a lot). Four out of nine sites indicated their team was highly engaged in the process, four suggested some, and partner involvement was lower than expected in Ghent.

**The Extent of Site-specific Suggestions and Work Reflected in the Core Draft Vignettes:**

Just over half of sites (5/9) indicated that they felt their work and feedback were sufficiently reflected in the subsequent versions of the vignettes. Three reported some level of suggestion-incorporation in the core vignette drafts. Again, Ghent reflected that their comments and team feedback was not sufficiently reflected in the version of vignettes ultimately deployed.

**Vignettes Piloting**

**Challenges During Piloting:**

Site team members from New Delhi and Kinshasa received feedback during piloting of the vignettes of additional ideas of vignette stories for adolescents. Due to limited time, site staff were not able to collect all these ideas in full. Ghent and Nairobi suggested the number of vignettes piloted were overwhelming and challenging for adolescents to stay engaged throughout the entire process. Shanghai reported adolescent boys initially had a challenging time understanding the parts of vignettes that required them to project themselves into protagonists’ perspectives, however they were able to comprehend the materials with explanations from interviewers.

All sites consistently reported that younger adolescents (10 – 12 years old) had difficulties in responding to vignette questions with one exception of New Delhi, where staff did not observe such difficulties.
